# The spontaneous self-assembly of a molecular water pipe in 3D space

**DOI:** 10.1107/S2052252522003396

**Published:** 2022-04-27

**Authors:** Ian R. Butler, Daniel M. Evans, Peter N. Horton, Simon J. Coles, Stewart F. Parker, Silvia C. Capelli

**Affiliations:** a Department of Chemistry, Bangor University, Bangor, Gwynedd LL57 2UW, United Kingdom; b EPSRC National Crystallography Service, School of Chemistry, Faculty of Engineering and Physical Sciences, University of Southampton, Southampton SO17 1BJ, United Kingdom; c ISIS Neutron and Muon Source, STFC Rutherford Appleton Laboratory, Harwell Science Campus, Didcot OX11 0QX, United Kingdom

**Keywords:** self-assembly, neutron diffraction, water pipes, biomimetic artificial water channels, TMP, crystal generation, X-ray diffraction

## Abstract

The self-assembly and self-organization of water molecules are relevant in many fields of research. When water spontaneously reacts with 2,2,6,6-tetra­methyl­piperidine (TMP) to form colourless and crystalline discrete needles, only in the exact ratio of 2:1, it is important to understand the phenomenon. Single-crystal X-ray and neutron diffraction data have unveiled that TMP self-assembles around columns of water molecules, and as such, the resulting adduct may be described as a series of molecular water pipes.

## Abstract 2

When an exact 2:1 ratio of water and 2,2,6,6-tetra­methyl­piperidine is mixed in a sealed vessel at room temperature, the formation of crystals is immediately observed. Single-crystal X-ray and neutron diffraction show that the phenomenon can be described as the spontaneous self-assembly of a 3D water pipe from its components.

## 1. Introduction

Water, the oxidation product of the most abundant element in the universe, is truly a primal molecule and its apparent structural simplicity belies its unique complex physical properties, which embed every aspect of life on our planet (Wiggins, 1990 ▸; Denny, 1993 ▸; Ball, 2008*a* ▸; Lynden-Bell *et al.*, 2010 ▸). From biological functions mediated by molecular-scale interactions in water (Ball, 2008*b* ▸), to water clusters in mixed planetary ices (Plattner *et al.*, 2010 ▸), the role of water molecules is fundamental but still not completely understood. Hydrogen bonding is clearly key to the unique properties of water and this gives rise to localized structural organizations of various types (Jeffrey, 1997 ▸; Bagchi, 2013 ▸; Li & Wu, 2015 ▸). Recently, new assemblies of water molecules, both in confined spaces (Radha *et al.*, 2016 ▸; Barsotti *et al.*, 2016 ▸; Kohonen & Christenson, 2000 ▸) and intracellular condensates (Jawerth *et al.*, 2020 ▸), have been reported, as well as details of capillary condensation under atomic scale confinement (Yang *et al.*, 2020 ▸; Fisher *et al.*, 1981 ▸).

Of all the mechanisms of water interactions, the transport of water through biological membranes has always attracted a lot of interest. In this area, aqua­porins (AQPs) are well known for the highly selective channels that, via single-file water wires, allow the transport of water at high speed (Heymann *et al.*, 1998 ▸; Maurel *et al.*, 2015 ▸; Luang & Hrmova, 2017 ▸). The specific interactions of water with the walls of the pores in AQPs is considered the term of reference for the development of biomimetic artificial water channels (Barboiu & Gilles, 2013 ▸, Barboiu, 2016 ▸). After showing that a high-flow-rate water channel can be constructed in lipid bilayer membranes from artificial stacks of four imidazoles (called I-quartets) and two water molecules (Le Duc *et al.*, 2011 ▸), additional studies were directed at understanding the interaction between dipolar orientation of the water molecules and the chirality of the walls and their effect on the mobility of the single-file water wires (Barboiu, 2012 ▸; Licsandru *et al.*, 2016 ▸; Kocsis *et al.*, 2018 ▸). The observation that the water channels formed by peptide-appended pillar[5]arenes (PAPs) can self-assemble into arrays in membranes (Shen *et al.*, 2015 ▸) has opened the possibility of engineering water channels for specific applications (Di Vincenzo *et al.*, 2021 ▸).

In this work, we focus our attention on the serendipitous discovery that an exact 2:1 ratio of water and 2,2,6,6-tetra­methyl­piperidine (TMP) self-assembles into a 3D structure that exhibits molecular water channels.

## 2. Sample preparation and preliminary characterization

TMP and its derivatives are simple molecules with a variety of interesting properties. Prominent derivatives are in the nitro­syl radical molecule TEMPO (2,2,6,6-tetra­methyl­piperidinyloxo), which is used in organic oxidations (Bartelson *et al.*, 2016 ▸), but TMP is also important in frustrated Lewis pair catalysis (Chernichenko *et al.*, 2012 ▸; Jupp & Stephan, 2019 ▸) and in research into adducts of CO_2_ (Ashley *et al.*, 2009 ▸) as metal-free reduction catalysts (Blondiaux *et al.*, 2014 ▸). During our recent synthetic work using TMP as a base for the selective lithium-assisted deprotonation of ferrocene derivatives (Butler *et al.*, 2020 ▸; Butler, 2021 ▸), the formation of a by-product, which appeared as a translucent aggregate of crystals, was observed in the vacuum manifold. On analysis of these crystals by NMR it appeared to be simply TMP, since the spectra were almost identical. However, on X-ray crystallographic analysis this was shown to be an adduct composed of water and TMP in the exact ratio of 2:1. In order to understand whether this was a simple crystallization of TMP with solvation water, additional experiments in the same condition but at different stoichiometric ratios from 90:10 to 10:90 were performed, but crystallization did not occur at any other ratio. We then made the compound by mixing water with TMP in the appropriate stoichiometry and the crystals formed from the two liquids on mixing. We prepared crystals in this way from both H_2_O and D_2_O. What really stood out as unusual behaviour was that when this compound was put in a stoppered vial, crystals would disappear and reform elsewhere in the vial according to where the vial was placed. Additional testing was carried out by applying gentle heating to one side of the vial and allowing it to stand, and the same phenomenon was observed again: some crystals melted or sublimed and recrystallized on a different surface of the vial. As the vial is sealed, we could think of this stoichiometric adduct as a binary system at its triple point, where the three different phases (solid, liquid and gas) coexist and a minor change in temperature can move the equilibrium between the phases. After all these observations, it was clear that we were dealing with a system in which the reagent in excess acted as a solvent for the other, giving rise to a crystalline structure that self-assembled from its constituent parts (see Figs. S1–S3 of the supporting information and the real-time supporting video file of the evolutions of the crystal in the sealed vial).

## 3. Structural analysis

**Figure 1:**
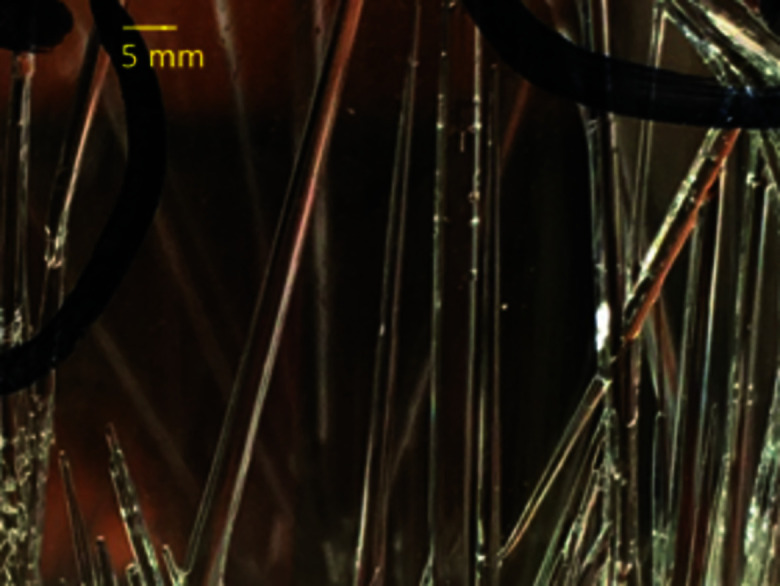
Rod-like crystals of the water–TMP complex as grown in the sealed vial.

The crystallographic structure of the water–TMP adduct was initially determined by single-crystal X-ray diffraction at 100 K, and subsequently by single-crystal neutron diffraction at 100 and 10 K. An actual image of the crystals showing their rod-like morphology is given in Fig. 1 ▸, while further images are shown in Fig. S4. Overall, the long tube-like crystals (rods) mirror the internal structure of the compound and can be described as a pseudo-tetragonal arrangement of TMP molecules that host, in the middle, a channel filled with water molecules, the whole assembly held together by hydrogen-bonding interactions. X-ray diffraction analysis at 100 K (see Fig. S5) showed that the structure has *P* 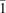 symmetry with centres of inversion covering all the body-centred and face-centred cubic positions in the unit cell. The asymmetric part of the unit cell contains two TMP molecules and four water molecules, of which only the oxygen atoms could be located (see Figs. S6 and S7).

In order to better understand the role of water and hydrogen bonding in assembling and stabilizing the structure, single-crystal neutron diffraction was performed at 100 and 10 K. Time-of-flight Laue diffraction data were collected with the single-crystal diffractometer SXD (Keen *et al.*, 2006 ▸) at the ISIS spallation neutron source using polychromatic radiation with λ in the range 0.3–8 Å (for the experimental method, see the supporting information).

**Figure 2:**
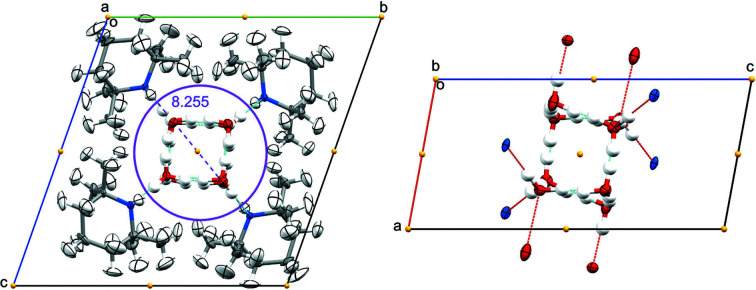
Structure of the water–TMP complex at 100 K from neutron-diffraction data: (*a*) a view down the **a** crystallographic axis, and (*b*) a view down the **b** crystallographic axis showing only the water cluster and the hydrogen bonding it forms. Ellipsoids are represented at 50% probability level; the purple circle indicates a diameter of ∼8.2 Å and the yellow dots indicate the positions of the centres of inversion in the unit cell.

The neutron-diffraction data collected on the TMP–H_2_O complex at 100 K confirms the X-ray structure and reveals positional disorder of the hydrogen atoms in the water molecules. The unit cell contains four molecules of TMP and eight water molecules symmetrically arranged around the inversion centre at the middle point of the unit cell, with the nitro­gen atoms of the piperidine ring pointing towards the inversion centre and the oxygen atoms of the water molecules sitting at the vertices of a distorted cube. Quartets of TMP molecules pile up along the direction of the crystallographic **a** axis, forming the walls of a channel of diameter ≃ 8.2 Å, which is filled with water molecules anchoring onto the walls via hydrogen bonds to the nitro­gen atoms of the piperidine ring. Additional hydrogen-bonding interactions between water molecules of the adjacent unit cells allows the expansion of the water pipe in the direction of the channel (see Fig. 2 ▸). The layout of the TMP molecules, giving rise to a water channel, is quite remarkably similar to the stack of imidazole molecules in the artificial water channel structure studied by Le Duc *et al.* (2011 ▸). The main difference is the dimension of the water channel, which in the case of the imidazole structure is ∼2.6 Å, allowing only a single-file water wire to sit inside the channel, while in the water–TMP structure the larger diameter allows the formation of a cubic octameric water cluster of *C_i_* symmetry.

**Figure 3:**
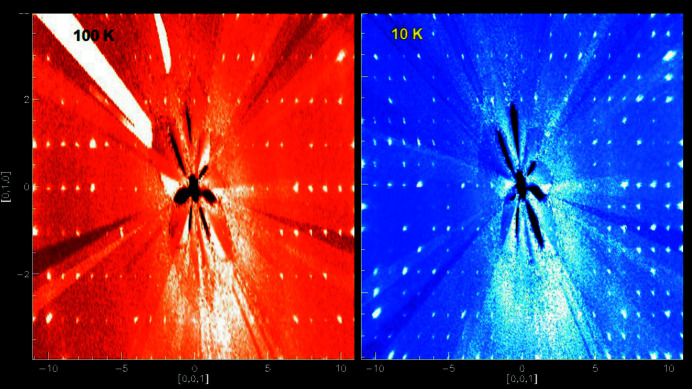
Comparison of reciprocal-space plots of the (0*kl*) plane at 100 K (left, red) and 10 K (right, blue), showing additional horizontal lines of reflections at 10 K, corresponding to a doubling of the **b** cell axis.

**Figure 4:**
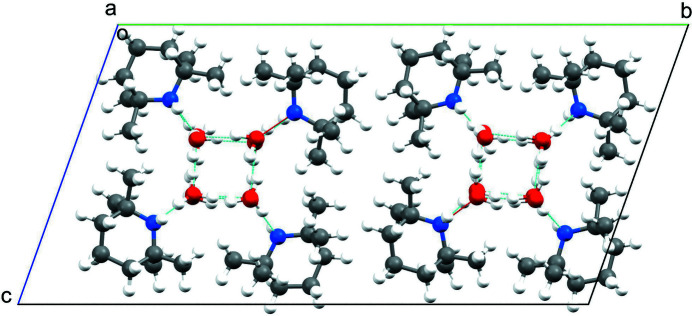
Structure of the water–TMP complex at 10 K from neutron-diffraction data, viewed down the **a** crystallographic axis, showing the two water channels.

The unit-cell parameters used to describe the structure at 100 K were unable to index all the Bragg peaks at 10 K. A reciprocal-space plot of the data showed additional lines of reflections in the *k* direction (see Fig. 3 ▸), corresponding to a doubling of the **b**-axis length and consequently of the unit-cell volume. The 10 K structure has the lowest symmetry (*P*1), and the unit cell contains eight TMP molecules and 16 water molecules in an arrangement very similar to that of the 100 K structure, with two independent water channels each surrounded by four TMP molecules forming the walls of the channels (see Fig. 4 ▸).

Although the arrangement of the oxygen atoms is still forming a distorted cube in each of the water channels, the corresponding O…O interatomic distances and angles in the cubes are slightly different, and also the distribution of the disordered hydrogen atoms is not the same in the two channels.

**Figure 5:**
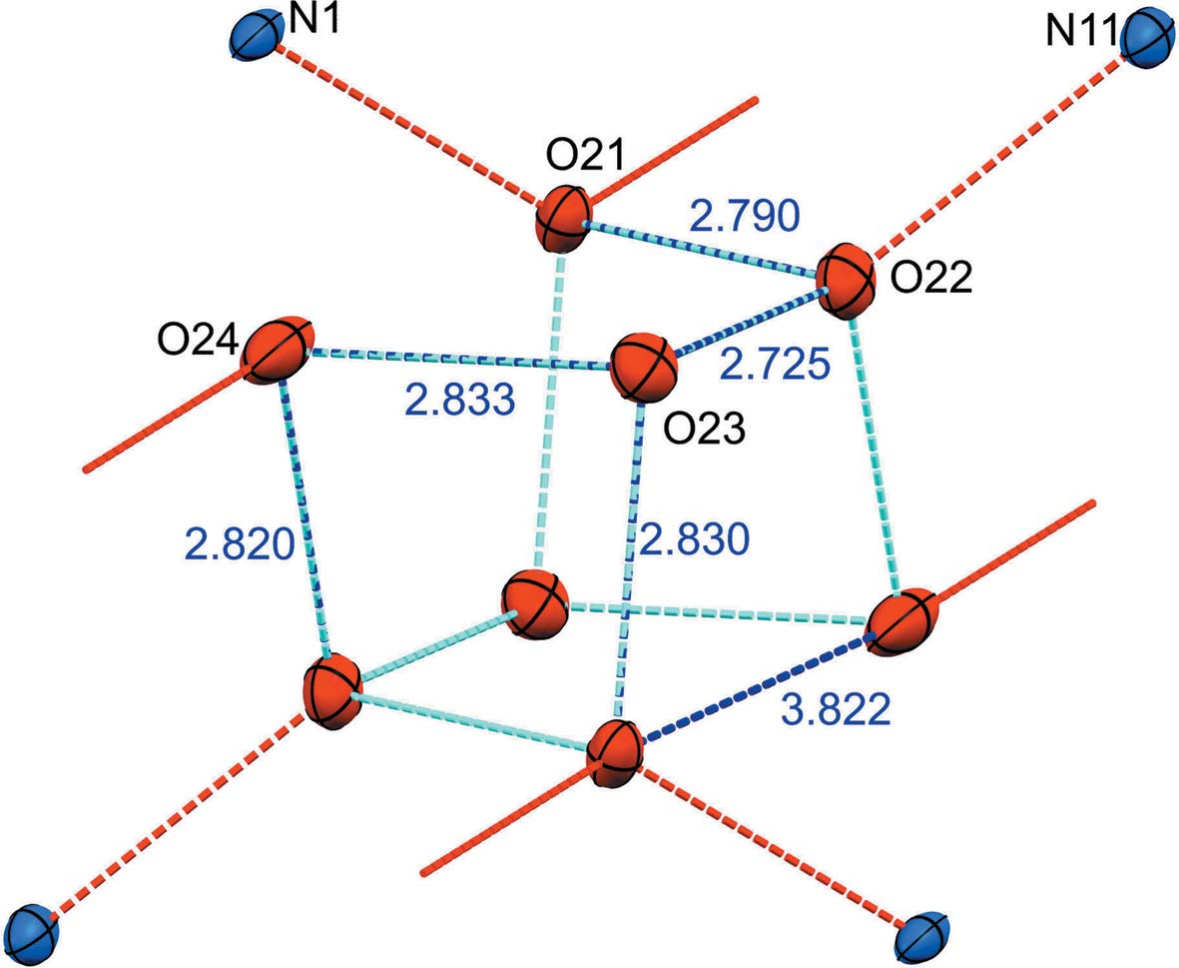
Pseudo-cubic arrangement (*C_i_* symmetry) of the oxygen atoms of the water molecules inside the channel at 100 K. The red dotted lines indicate the hydrogen bonding to the nitro­gen atoms of the TMP walls and to the oxygen atoms in neighbouring octamers along the channels.

The overall comparison of the structures at 100 and 10 K points towards a completely dynamic system, kept together by hydrogen-bonding interactions between the TMP and water moieties, and between the water molecules themselves (see Figs. S8–S10 for a detailed description of the water channels and hydrogen bonding). It is interesting to note the distorted-cubic arrangement of the oxygen atoms of the water molecules in the unit cell (see Fig. 5 ▸): at both temperatures, ten out of the 12 edges of the cube are directions of strong hydrogen bonding with O…O interaction distances in the range 2.68 (1)–2.83 (1) Å, while the remaining two edges represent oxygen atoms that are ∼3.73 (1)–3.87 (1) Å far apart. Within the channels, the octameric water clusters pile up as cubes stacked face on face and held together by hydrogen bonds to the nearby clusters along the channel direction, reminiscent of the arrangement of the most stable configurations theoretically calculated for water clusters with 12, 16 and 20 molecules (Lee *et al.*, 1995 ▸).

Bond distances do not show drastic changes on lowering the temperature but there are adjustments to the short-range interactions: for example, the N1 atom, which is an acceptor of a hydrogen bond from O21 at 100 K, becomes at 10 K also a donor with the H1 hydrogen bonding to O4 (similarly for O9…N1F…O12 in the second channel).

Although theoretical studies of water clusters have been ongoing for more than 50 years (Del Bene & Pople, 1970 ▸), the cubic octamer water cluster (Kistenmacher *et al.*, 1974 ▸) has appeared in the literature mainly as a theoretically possible geometrical arrangement in the context of computational energy calculations to establish the most stable form of water clusters or the best computational method to describe them (Lee *et al.*, 1995 ▸; Estrin *et al.*, 1996 ▸; Maheshwary *et al.*, 2001 ▸; Belair & Francisco, 2003 ▸; Day *et al.*, 2005 ▸). Spectroscopic studies have proven the existence of *S*_4_ and *D*_2*d*_ symmetry for cubic water clusters in the gas phase (Buck *et al.*, 1998 ▸; Cole *et al.*, 2016 ▸; Li *et al.*, 2020 ▸), but only a limited number of cubic arrangements of water molecules have been found in crystalline materials.

Extensive work has been carried out to classify patterns of water clusters in organic molecular crystal hydrates (Infantes & Motherwell, 2002 ▸; Infantes *et al.*, 2003 ▸; Mascal *et al.*, 2006 ▸) and to establish a correlation between the donor/acceptor properties of the functional groups in the organic moiety and the likelihood of hydrates formation (Infantes *et al.*, 2007 ▸). As a result, tools are now available to every user of the Cambridge Structural Database (CSD) (Groom *et al.*, 2016 ▸) to search for extended water motifs in crystalline hydrates. A search of the CSD gives, to date, 96 structures in which there is a ‘water cube’ motif, only three of which are completely organic, *i.e.* composed only of C, H, O and N (refcodes DOCYID, DOXVUH and TAHREZ) (Kamitori *et al.*, 1986 ▸; Taga *et al.*, 1986 ▸; Ashton *et al.*, 1996 ▸), with all the others being either metal–organic complexes or salts.

The DOXVUH structure, *i.e.* 5,14-di­acetyl-9b,9c,18b,18c-tetra­hydrotetrabenzo[*b,b′,f,f′*]cyclo­buta[1,2-*d*:3,4-*d′*]bis(azepine) hydrate (Taga *et al.*, 1986 ▸), crystallizes in the *R* 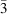 space group and shows an hexagonal arrangement of the organic moiety forming a channel of diameter ≃ 10.7 Å along the [111] direction, in which a chain of water cubes piles up along the body diagonal. In this direction the cubes do not share vertices but the oxygen atoms of two adjacent cubes are ∼3.015 Å apart. The O…O distances within the water cube range from 2.85 to 3.12 Å while the O…O separation between the oxygen atoms in the cube interacting with those in the wall of the channel is ∼2.99 Å.

Amongst the metal–organic compounds, the crystalline framework material [V(phen)_2_SO_4_]_2_O(H_2_O)_4_ (phen = 1,10-phenanthroline) (Doedens *et al.*, 2002 ▸) displays an octameric cluster of water molecules that is inversion symmetric (*C_i_*) and shows the same pattern of ten strong hydrogen-bonded edges of the cube with distances ranging from 2.76 to 2.93 Å and the remaining two, called ‘open edges’, with distances between the oxygen atoms of 3.92 Å. The pseudo-cubic arrangement of the oxygen atoms is remarkably similar to one of the water–TMP adducts, but in that case, the structure has no channels and the clusters are joined by sulfate bridges. Another cubic arrangement of water molecules, fairly similar to the one reported here, was observed in the 3D supramolecular network of composition [Cu(4-di­methyl­amino­pyridine)_4_]Cl_2_·2.5H_2_O (Seth, 2014 ▸). In this case, the cube is more regular, showing O⋯O separation distances ranging from 2.74 to 3.01 Å and no open edges. Here again, the structure does not show channels, possibly because the water cubes form a chain along the threefold axis of the cube (body diagonal), which does not allow hydrogen bonding between nearby cubes. The metal–organic framework (MOF) *catena*-[(μ-5-meth­oxy­benzene-1,3-di­carboxyl­ato)aquacopper(II) monohydrate] (Garai *et al.*, 2017 ▸) shares two remarkable similarities with the water–TMP complex: (i) the final MOF seems to self-assemble from a stoichiometric amount of its constituent parts, *i.e.* when crystals of the corresponding metal–organic polyhedra are treated with a specific amount of water; and (ii) the water cubes are stacked face on face in a square channel (diameter ≃ 9 Å) formed by the MOF.

In the water–TMP complex, the high degree of disorder at both temperatures, the fact that at 10 K the two water channels show a different distribution of the water hydrogen atomic positions, and the fast degradation of the crystal as soon as they are out of the vial and exposed to air at room temperature, suggest a continuous flow of water molecules in the channels. The anisotropic displacement parameters of the water oxygen atoms at 100 K are fairly similar in the three crystallographic directions and only for O24 is the ellipsoid slightly elongated along the channel direction. This could suggest that the mechanism of water transport could actually occur via proton transfer with a Grotthuss-type mechanism (Grotthuss, 1806 ▸), as already suggested for explaining water transport in AQPs (Fujiyoshi *et al.*, 2002 ▸) and in other molecular solids (Capelli *et al.*, 2013 ▸).

A summary of the details of the refinements including final cell parameters and agreement factors is given in Table S1 of the supporting information, while details of the hydrogen bonding between the water molecules and the TMP frame are given in Table S2.

## 4. Vibrational spectroscopy

**Figure 6:**
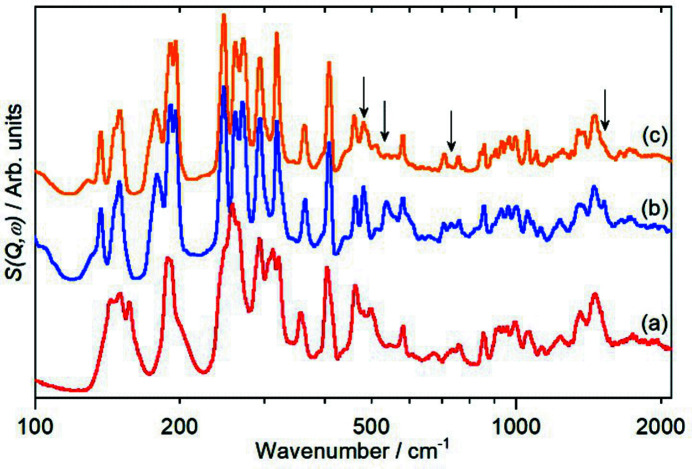
INS spectra at 10 K of (*a*) TMP, (*b*) TMP–H_2_O and (*c*) TMP–D_2_O. The arrows indicate positions where there is a difference between the H_2_O and D_2_O complexes. Note that the *x* axis is on a log_10_ scale.

The TMP/water complex has also been investigated by inelastic neutron scattering (INS) spectroscopy (Mitchell *et al.*, 2005 ▸). INS is a form of vibrational spectroscopy where the spectra are dominated by modes that involve hydrogen motion. Fig. 6 ▸ compares the INS spectrum of TMP with that of the TMP:H_2_O and TMP:D_2_O complexes. The 100–400 cm^−1^ region is where the TMP skeletal deformations and the methyl torsions occur. It can be seen that these are modified to some degree on complex formation, but that they are identical for both the H_2_O and D_2_O complexes. Between 400 and 2000 cm^−1^ the spectrum of TMP is largely unchanged on complexation. However, there are some differences (arrowed in Fig. 6 ▸) between the H_2_O and D_2_O complexes. A periodic-DFT calculation of the TMP–H_2_O complex (see Fig. S11) shows that the modes at 482, 534 and 733 cm^−1^ are water vibrational modes, they do not appear in the TMP–D_2_O complex’s spectrum because they have been downshifted owing to the mass increase and they have diminished in intensity because the neutron-scattering cross section of deuterium is only ∼5% that of ^1^H. The mode at 1526 cm^−1^ is an in-plane N–H bending mode, this exchanges with D_2_O to generate an N–D species that is not detected.

We also studied the temperature dependence of the spectra in the hope that it may be possible to see ‘melting’ of the entrained water. Unfortunately, as Figs. S12–S14 show, this did not occur, and both the TMP–H_2_O and TMP–D_2_O complexes behave the same as the parent TMP, with all of the changes with temperature being due to the increasing Debye–Waller factor. These are completely reversible, as shown by the superposition of the initial spectrum of the TMP–D_2_O complex with the spectrum after it had been heated to 220 K [Fig. S14(*a*)].

## 5. Conclusions

The serendipitous discovery of an unexpected by-product in a vacuum line has been shown to be a spontaneous self-assembly of water and TMP molecules in the exact ratio 2:1. The novel material has been studied by X-ray and neutron diffraction and inelastic neutron spectroscopy to reveal that the structure consists of quartets of TMP molecules stacked along the **a** crystallographic axis, forming channels filled with pseudo-cubic octameric water clusters piled face on face. The disorder of the hydrogen atoms of the water molecules is a strong indication that the system is completely dynamic. An octameric water cluster is quite unusual in crystalline structures, especially when it develops in a columnar arrangement, and its presence in this new structure can help in the understanding and design of artificial water pipes.

## 6. Related literature

The following references are cited in the supporting information for this article: Dassault Systeme (2021) ▸, Dolomanov *et al.* (2009) ▸, Gutmann (2017) ▸, Milman *et al.* (2009) ▸, Perdew *et al.* (1996) ▸, Pinna *et al.* (2018) ▸, Ramirez-Cuesta (2004) ▸, Refson *et al.* (2006) ▸, Rigaku (2013 ▸, 2017 ▸), Sheldrick (2008 ▸, 2015*a* ▸,*b* ▸) and Tkatchenko & Scheffler (2009) ▸.

## Supplementary Material

Crystal structure: contains datablock(s) tmp_100_3d, tmp_10_3d, IRBpipe1. DOI: 10.1107/S2052252522003396/yc5037sup1.cif

Supporting information. DOI: 10.1107/S2052252522003396/yc5037sup2.pdf

Real-time video of the crystallisation process. DOI: 10.1107/S2052252522003396/yc5037sup3.avi

The neutron diffraction data: https://doi.org/10.5286/ISIS.E.RB1720101

The INS experimental data: https://doi.org/10.5286/ISIS.E.RB1720111

CCDC references: 2012977, 2083914, 2083915
