# Supplementary material for: The spontaneous self-assembly of a molecular water pipe in 3D space
Source: IUCrJ. 2022 Apr 27;9(Pt 3):364–9. doi: 10.1107/S2052252522003396 (PMC9067119; doi:10.1107/S2052252522003396)
Supplement: Supplementary file 2 [file m-09-00364-sup2.pdf]

IUCrJ

Volume 9 (2022)

Supporting information for article:

The spontaneous self-assembly of a molecular water pipe in 3D space

Ian R. Butler, Daniel M. Evans, Peter N. Horton, Simon J. Cole, Stewart F. Parker and Silvia C. Capelli

Table of Contents

S1. Experimental Methods

S1. Tables

Table S1. Summary of crystallographic details from the diffraction experiments.
Table S2. Hydrogen bonding interaction distances for the in the water-TMP structure at 100 and 10K.
Table S3. CIF file of the geometry optimised structure of the water-TMP crystal with full occupancy of all atoms.

S2. Figures

Figure S1: Stacked ^{1}H NMR spectra of TMP:D_2O ratios of 100:0 to 10:90.
Figure S2: Stacked ^{1}H NMR spectra of TMP:D_2O ratios of 100:0 to 10:90, with expansion to highlight resonance shifts.
Figure S3: ^{1}H NMR spectrum of 2,2´,6,6´-tetramethylpiperidine for reference.
Figure S4: Photographs of the TMP:H_2O complex showing the rod-like morphology.
Figure S5: Structure of the TMP:H_2O crystal at 100 K (X-ray data).
Figure S6: Molecular views of the water channel showing the interaction between the water molecules and external TMP molecules (X-ray data, 100 K).
Figure S7: Molecular views of the water channel using space filling models for the water molecules (X-ray data, 100 K).
Figure S8: Hydrogen bonds between water and TMP in the crystal at 100 K (neutron data).
Figure S9: Extended structure showing a view down the molecular water pipe “tubes” (neutron data, 100 K).
Figure S10: Side view of the crystalline packing showing individual molecular “water pipes” (neutron data, 100 K).
Figure S11: Comparison of the calculated and observed (10 K) INS spectra of TMP:H_2O.
Figure S12: Evolution of the INS spectrum of TMP with temperature.
Figure S13: Evolution of the INS spectrum of TMP:H_2O with temperature.
Figure S14: Evolution of the INS spectrum of TMP:D_2O with temperature.

S3. Experimental methods

S3.1. Original isolation of compounds.

In the original work an aggregate of colorless crystals were removed from the glass line of a high vacuum manifold when drying fresh research samples of 1,1',2,2'-tetrabromoferrocene when 2,2´,6,6´-tetramethylpiperidine was used as a reactant in the synthesis of 1,1'-dibromoferrocene.

S3.2. Preparation of the 2,2´,6,6´-tetramethylpiperidine-water adduct

The title compound was prepared by mixing fresh 2,2´,6,6´-tetramethylpiperidine with water in the exact stoichiometric ratio of 2:1. In the attached video, water (6.4 mL, i.e. 355 mmol) was added to 2,2´,6,6´-tetramethylpiperididne (25 g, i.e. 177 mmol) in a sealed container. After 10 minutes, the adduct has crystallized and sublimed (100%).

S3.3. NMR experiments

NMR experiments were performed using a Bruker Avance instrument operating at 400 MHz for proton using $CDCl_3$ as solvent. Solutions in the following stoichiometric ratios were prepared: D_2O: TMP 10,0; 9,1; 8,2; 7,3; ,6,4; 5,5; ,4,6; 3,7; ,2, 8; 1,9; 0,10. These were diluted to a 1% concentration in $CDCl_3$.

S3.4. X-ray diffraction experiment

A suitable colourless blade crystal (0.180 × 0.070 × 0.020) mm^3 was selected and mounted on a MITIGEN holder in oil on a Rigaku FRE+ diffractometer equipped with HF Varimax confocal mirrors, an AFC12 goniometer and HG Saturn 724+ detector. The crystal was kept at T = 100(2) K during data collection. Data were measured using profile data from ω-scans using MoKα radiation. Cell determination and data collection were carried out using CrystalClear (Rigaku, 2013). With the data reduction, cell refinement and absorption correction using CrystalisPro (Rigaku, 2017). Using Olex2 (Dolomanov *et al.*, 2009), the structures were solved with the ShelXT (Sheldrick, 2015a) structure solution program and the models were refined with version 2014/7 of ShelXL (Sheldrick, 2015b) using Least Squares minimisation. All non-hydrogen atoms were refined anisotropically. The position of hydrogen atoms attached to carbon atoms were calculated geometrically and refined using the riding model with the rest found from the difference map and their positions allowed to be freely refined. Crystal data are given in Table S1.

S3.5. Neutron diffraction experiments

In order to minimize sublimation during manipulation, the vial containing the crystals was opened in a glovebox under nitrogen atmosphere on a metallic working surface kept at 5 °C by means of an external water bath. A suitable crystal of size (18 x 4 x 1) mm^3 was extracted from the vial and sealed in a 6 mm diameter quartz capillary with a double layer of Parafilm and aluminium tape. The quartz capillary and all the tools used in handling were left in contact with the metallic surface for about 15 min before using them in order to thermalize them and minimize the possible damage to the crystal. The sealed quartz capillary containing the crystal was left to thermalize in the glovebox for additional 15 min and then mounted at the end of a centre stick to be inserted in a top-loading cryo-refrigerator already mounted on the SXD instrument at the ISIS Neutron and Muon Source and kept at 100 K. Diffraction data were measured at 100 and 10 K, using the time-of-flight Laue method, in a series of 8 orientations around the vertical axis of the instrument with a counting time of ~8 h each at 100 K, while 9 orientations with a 4 h counting time were used at 10 K. The data at 100 K were initially indexed with the unit cell available from the 100 K X-ray measurement but at 10 K the cell parameters were derived by measuring the positions of reflections in a reciprocal space plot. Bragg intensities at the two temperatures were extracted from all data using the 3D-profile fitting method implemented in the SXD2001 software (Gutmann, 2017) and corrected for the Lorentz effect. No absorption correction for the shape of the crystal was applied. Final cell parameters at each temperature were refined against the fitted positions of the Bragg peaks after 3D profile integration. The starting model for structural refinement at 100 K was based on the atomic coordinates of the non-hydrogen atoms from the X-ray structure at room temperature, while all hydrogen atoms were located from neutron Fourier difference maps. The structure at 10 K was solved ab initio by direct methods using ShelXS (Sheldrick, 2008). Both structures were refined by full matrix least squares on F^2 using ShelXL (Sheldrick, 2015b). At 100 K the data-to-parameters ratio allowed the anisotropic refinement of all atoms in the TMP moiety, including the hydrogens, while for the water molecules only the oxygen atoms were refined anisotropically. The hydrogen atoms in the water molecules showed positional disorder with non-integer occupation factors, except for the two hydrogens involved in the hydrogen bonding with the TMP molecules. Disordered hydrogen atoms were refined isotropically with a group thermal parameter but their occupation factors were left free to refine with no overall restraints. The O-H distances in the water molecules were all restrained to be the same without imposing a target value. At 10 K the increased number of independent molecules in the unit cell (Z'=8) would not allow an anisotropic structural refinement for the available number of observations, therefore all atoms were refined isotropically with group thermal parameters. The water molecules in the two independent channels showed a different distribution of the disordered hydrogen atoms, therefore the O-H distances were restrained to be the same for all water molecule only within the same channel, but with no specific target value. Occupation factors were set to unity for all hydrogens involved in strong hydrogen bonding, while it was left free to refine for the positional

disordered atoms. To further reduce the number of parameters, the hydrogen atoms in the -CH_3 groups in the TMP moiety were refined at idealised atomic positions and all atoms were refined with group thermal parameters. Crystal data are given in Table S1 while details of the hydrogen bonding parameters are given in Table S2. All experimental data can be accessed at DOI: **10.5286/ISIS.E.RB1720101**.

S3.6. CCDC

All crystallographic data reported in this paper have been deposited with the joint Cambridge Crystallographic Data Centre and Fachinformationszentrum Karlsruhe database service. The data are provided free of charge to anybody who require them via www.ccdc.cam.ac.uk/structures.

Reference numbers 2012977, 2083914 and 2083915 refer respectively to the 100 K X-ray data, the 100 K neutron data and the 10 K neutron data.

S3.7. Inelastic neutron scattering (INS) spectroscopy experiments

Samples of TMP-H_2O and TMP-D_2O were prepared immediately before the measurement by mixing stoichiometric quantities of TMP (Aldrich, >99%) and distilled water or D_2O (Aldrich, 99.9% D) and loading the sample into In-sealed, thin-walled, flat-plate Al cells. INS spectra were measured using TOSCA (Pinna et al., 2018) at the ISIS Neutron and Muon Source at temperatures in the range 10 – 220 K. All experimental data can be accessed at DOI: **10.5286/ISIS.E.RB1720111**.

S3.8. Computational studies

Dispersion corrected periodic density functional theory calculations were carried out on the complete unit cell using CASTEP (version 17.21) (Clark et al, 2005; Refson et al., 2006). Exchange and correlation were approximated using the PBE (Perdew et al., 1996) functional with the Tkatchenko-Scheffler dispersion correction scheme (Tkatchenko & Scheffler, 2009) within the generalized gradient approximation. The plane-wave cut-off energy was 1830 eV. Brillouin zone sampling of electronic states was performed on a 6 x 2 x 4 Monkhorst-Pack grid (24 k-points). The equilibrium structure was obtained by BFGS geometry optimization after which the residual forces were converged to ±0.0095 eV Å 1. Phonon frequencies were obtained by diagonalization of dynamical matrices computed using density-functional perturbation theory (Milman et al., 2009). The atomic displacements in each mode that are part of the CASTEP output, enable visualization of the modes to aid assignments in Materials Studio (Dassault Systeme, 2021) and are what is required to generate the INS spectrum using the program ACLIMAX (Ramirez-Cuesta, 2004). It is emphasised that for the calculated spectra shown, the transition energies have not been scaled.

The initial structure used for the calculations was the 10 K neutron diffraction structure. However, this has several atoms with partial occupancies. Based on the surrounding structure, the most "chemically reasonable" structure was selected that had full occupancy of all the atoms. The cif file of the geometry optimised structure is given in Table S3. The phonon calculation resulted in one small imaginary mode (-24 cm^{-1}), which the mode animation shows to be a librational mode of TMP, so does not influence the spectrum. Figure S10 shows a comparison of the observed and calculated INS spectra, the agreement is sufficiently good as to allow mode assignments to be made.

S3.9. References

Clark, S.J., Segall, M.D., Pickard, C.J., Hasnip, P.J., Probert, M.J., Refson K., & Payne, M.C. (2005). *Z. Krist.*, **220**, 567-570.

Dassault Systeme, (2021) Molecular modelling simulation software: Biovia-Material-Studio

Dolomanov, O.V., Bourhis, L.J., Gildea, R.J., Howard, J.A.K., and Puschmann, H., (2009). *J. Appl. Cryst.*, **42**, 339-341.

Gutmann, M.J., (2017). *Nucl. Instrum. Meth.* **A848**, 170-173.

Milman, V., Perlov, A., Refson, K., Clark, S.J., Gavartin J. & Winkler, B. (2009) *J. Phys.: Condens. Matter*, **21**, 485404.

Perdew, J., Burke K. & Ernzerhof, M. (1996) *Phys. Rev. Lett.*, **77**, 3865.

Pinna, R.S., Rudić, S., Parker, S.F., Armstrong, J., Zanetti, M., Škoro, G., Waller, S.P., Zacek, D., Smith, C.A., Capstick, M.J., McPhail, D.J., Pooley, D.E., Howells, G.D., Gorini G. & Fernandez-Alonso, F. (2018). *Nucl. Instrum. Meth.*, **A896**, 68-74.

Ramirez-Cuesta, A. J. (2004). *Comput. Phys. Commun.*, **157**, 226-238.

Refson, K., Tulip P.R. & Clark, S.J. (2006). *Phys. Rev. B*, **73**, 155114.

Rigaku (2013) CrystalClear- SM Expert 3.1 b27.

Rigaku (2017) CrysAlisPro Software System, Rigaku Oxford Diffraction, Yarnton, Oxford, UK.

Sheldrick, G.M., (2008). *Acta Cryst.*, **A64**, 112-122.

Sheldrick, G.M., (2015a). Acta Cryst., **A71**, 3-8.

Sheldrick, G.M., (2015b). *Acta Cryst.*, **C71**, 3-8.

Tkatchenko A. & Scheffler, M. (2009) *Phys. Rev. Lett.*, **102**, 073005.

Table S1 Summary of crystallographic details from the diffraction experiments.

Crystal system	Triclinic	Triclinic	
Crystal size	0.18 x 0.07 x 0.02 mm^3	18 x 4 x 1 mm^3	
Radiation type	X-ray	Neutron	
Wavelength	Mo K_{α} (0.7073 Å)	White beam (0.3-8 Å)	
Temperature	100 K	100 K	10 K
Space group	$P\bar{1}$	$P\bar{1}$	P 1
Cell parameters	a = 6.5253(3) Å<br>b = 13.4883(5) Å<br>c = 14.2589(6) Å<br>α = 108.981(4) °<br>β = 99.087(4) °<br>γ = 91.859(3) °	a = 6.5456 (3) Å<br>b = 13.5305(5) Å<br>c = 14.2972 (6) Å<br>α = 108.79 (4) °<br>β = 99.27 (4) °<br>γ = 91.90 (3) °	a = 6.5173 (5) Å<br>b = 26.9954 (6) Å<br>c = 14.2406(4) Å<br>α = 109.07 (4) °<br>β = 98.93 (4) °<br>γ = 92.02 (3) °
Cell volume	1167.15(9) $Å^3$	1178.2(1) $Å^3$	2329.3(1) $Å^3$
Z, Z‘	4, 2	4, 2	8, 8
Total no. of reflections	19804	5091	9293
No of reflection with I/σ(I) > 4		5052	9242
$R(int)_{X\text{-}ray}$ or $R(sigma)_{neut}$	0.0403	0.0420	0.0566
Parameters/Restraints	270 / 0	621 / 78 [a)]	690 / 576 [a,b)]
R1, wR2, Goof (all data)	0.0711, 0.1270, 1.027	0.0920, 0.2383, 1.207	0.1046, 0.2645, 1.266
R1 after Fourier merging / No of unique reflections	0.0483 / 3967	0.0813 / 2790	0.1031 / 5407
Max resolution (Å)	0.77	0.48	0.27
Electron or Nuclear density Fourier difference residuals: $\Delta\rho_{max}$, $\Delta\rho_{min}$	0.313, -0.215	1.41, -1.41	3.33, -2.99
Extinction coeff.		0.0028(3)	0.0006(1)

a) All O-H distances in the solvent water molecules have been restrained to be the same without imposing a target value.

b) The hydrogen atoms of the CH_3 groups in the TMP moiety have been refined at idealised atomic positions with group isotropic thermal parameters.

Table S2 Hydrogen bonding interaction distances for the in the water-TMP structure at 100 and 10K.

H-bond	d(D-H) (Å)	d(H…A) (Å)	d(D…A) (Å)	angle(DHA) (°)	T (K)
O21-H21A...N1	0.976(9)	1.795(9)	2.772(6)	180(1)	100
O1-H11...N1	0.994(11)	1.85(2)	2.83(2)	171(3)	10
O9-H91...N1F	0.999(11)	1.77(2)	2.77(2)	177(3)	10
N1-H1...O24	1.025(17)	2.118(15)	3.083(8)	156(1)	100
N1-H1N…O4	0.87(5)	2.10(5)	2.96(3)	167(3)	10
N1F-H1NF...O12	1.27(4)	1.94(4)	3.08(3)	147(4)	10
O22-H22A...N11	0.984(9)	1.817(10)	2.800(7)	178(1)	100
O2-H21...N1G	0.997(11)	1.87(3)	2.84(3)	168(3)	10
O10-H101...N1E	1.001(11)	1.73(3)	2.73(2)	177(4)	10
O6-H61...N1A	0.995(11)	1.71(2)	2.70(2)	171(3)	10
O14-H141...N1C	1.004(11)	1.85(2)	2.85(2)	176(3)	10
O5-H51...N1B	0.994(11)	1.74(2)	2.72(2)	169(3)	10
O13-H131...N1D	1.003(11)	1.80(2)	2.79(2)	171(3)	10

Table S3 CIF file of the geometry optimised structure of the water-TMP crystal with full occupancy of all atoms.

```
data_TMP-H2O
_audit_creation_date              2020-04-30
_audit_creation_method            'Materials Studio'
_symmetry_space_group_name_H-M    'P1'
_symmetry_Int_Tables_number       1
_symmetry_cell_setting            triclinic
loop_
_symmetry_equiv_pos_as_xyz
  x,y,z
_cell_length_a                    6.5173
_cell_length_b                    26.9954
_cell_length_c                    14.2406
_cell_angle_alpha                 109.0690
_cell_angle_beta                  98.9330
_cell_angle_gamma                 92.0180
loop_
_atom_site_label
_atom_site_type_symbol
_atom_site_fract_x
_atom_site_fract_y
_atom_site_fract_z
_atom_site_U_iso_or_equiv
_atom_site_adp_type
_atom_site_occupancy
N1     N     0.46279   1.14118   0.27535   0.00040  Uiso   1.00
H2     H     0.34065   1.15889   0.30714   0.00900  Uiso   1.00
C3     C     0.44430   1.14580   0.17269   0.00245  Uiso   1.00
C4     C     0.27521   1.10461   0.09573   0.00245  Uiso   1.00
H5     H     0.12107   1.11692   0.11188   0.01690  Uiso   1.00
H6     H     0.28185   1.10518   0.01932   0.01690  Uiso   1.00
C7     C     0.29286   1.04902   0.09973   0.00245  Uiso   1.00
H8     H     0.43540   1.03353   0.07349   0.01690  Uiso   1.00
H9     H     0.16262   1.02216   0.04722   0.01690  Uiso   1.00
C10    C     0.29340   1.04847   0.20657   0.00245  Uiso   1.00
H11    H     0.31342   1.00870   0.20989   0.01690  Uiso   1.00
H12    H     0.14050   1.05860   0.22775   0.01690  Uiso   1.00
C13    C     0.46474   1.08786   0.28559   0.00245  Uiso   1.00
C14    C     0.37823   1.20100   0.18297   0.00245  Uiso   1.00
H15    H     0.22588   1.20576   0.20695   0.02140  Uiso   1.00
H16    H     0.36648   1.20779   0.11040   0.02140  Uiso   1.00
H17    H     0.49129   1.23160   0.23893   0.02140  Uiso   1.00
C18    C     0.65627   1.14120   0.13701   0.00245  Uiso   1.00
H19    H     0.77866   1.16766   0.19552   0.02140  Uiso   1.00
H20    H     0.64753   1.15218   0.06872   0.02140  Uiso   1.00
H21    H     0.70569   1.10130   0.11866   0.02140  Uiso   1.00
C22    C     0.41856   1.09483   0.39115   0.00245  Uiso   1.00
H23    H     0.41899   1.05713   0.40510   0.02140  Uiso   1.00
H24    H     0.26501   1.10948   0.39906   0.02140  Uiso   1.00
H25    H     0.53510   1.12314   0.44989   0.02140  Uiso   1.00
```

C26	C	0.68083	1.06695	0.27849	0.00245	Uiso	1.00
H27	H	0.71176	1.05270	0.20100	0.02140	Uiso	1.00
H28	H	0.69182	1.03377	0.30695	0.02140	Uiso	1.00
H29	H	0.80495	1.09776	0.32416	0.02140	Uiso	1.00
N30	N	0.13413	1.18670	-0.29588	0.00040	Uiso	1.00
H31	H	-0.00824	1.19983	-0.31402	0.00500	Uiso	1.00
C32	C	0.11297	1.12819	-0.34408	0.00245	Uiso	1.00
C33	C	0.00159	1.10120	-0.28298	0.00245	Uiso	1.00
H34	H	-0.16401	1.10913	-0.29234	0.01690	Uiso	1.00
H35	H	0.00357	1.05822	-0.31537	0.01690	Uiso	1.00
C36	C	0.09437	1.12118	-0.17052	0.00245	Uiso	1.00
H37	H	0.25475	1.10987	-0.15818	0.01690	Uiso	1.00
H38	H	0.00551	1.10224	-0.12985	0.01690	Uiso	1.00
C39	C	0.09115	1.18090	-0.12726	0.00245	Uiso	1.00
H40	H	-0.07274	1.19037	-0.13236	0.01690	Uiso	1.00
H41	H	0.16056	1.19587	-0.04645	0.01690	Uiso	1.00
C42	C	0.20341	1.21047	-0.18413	0.00245	Uiso	1.00
C43	C	-0.02202	1.11620	-0.44852	0.00245	Uiso	1.00
H44	H	-0.17678	1.13024	-0.44157	0.02140	Uiso	1.00
H45	H	-0.04273	1.07362	-0.48973	0.02140	Uiso	1.00
H46	H	0.04857	1.13552	-0.49420	0.02140	Uiso	1.00
C47	C	0.32775	1.10759	-0.35770	0.00245	Uiso	1.00
H48	H	0.41883	1.13244	-0.38675	0.02140	Uiso	1.00
H49	H	0.30820	1.06728	-0.41197	0.02140	Uiso	1.00
H50	H	0.42012	1.10662	-0.28716	0.02140	Uiso	1.00
C51	C	0.14248	1.26714	-0.15555	0.00245	Uiso	1.00
H52	H	0.21816	1.28929	-0.19518	0.02140	Uiso	1.00
H53	H	0.18841	1.28788	-0.07396	0.02140	Uiso	1.00
H54	H	-0.02697	1.26750	-0.17580	0.02140	Uiso	1.00
C55	C	0.44209	1.21192	-0.15483	0.00245	Uiso	1.00
H56	H	0.49482	1.17313	-0.15982	0.02140	Uiso	1.00
H57	H	0.49335	1.23801	-0.07637	0.02140	Uiso	1.00
H58	H	0.52269	1.22699	-0.20354	0.02140	Uiso	1.00
N59	N	0.58313	1.40902	-0.24400	0.00040	Uiso	1.00
H60	H	0.70595	1.39206	-0.27625	0.00900	Uiso	1.00
C61	C	0.60728	1.40504	-0.14070	0.00245	Uiso	1.00
C62	C	0.77428	1.44744	-0.06537	0.00245	Uiso	1.00
H63	H	0.92904	1.43623	-0.08255	0.01690	Uiso	1.00
H64	H	0.77219	1.44722	0.01165	0.01690	Uiso	1.00
C65	C	0.74743	1.50254	-0.07061	0.00245	Uiso	1.00
H66	H	0.60370	1.51686	-0.04392	0.01690	Uiso	1.00
H67	H	0.87550	1.53055	-0.01911	0.01690	Uiso	1.00
C68	C	0.74166	1.50270	-0.17802	0.00245	Uiso	1.00
H69	H	0.89484	1.49359	-0.19985	0.01690	Uiso	1.00
H70	H	0.71587	1.54211	-0.18213	0.01690	Uiso	1.00
C71	C	0.57280	1.46193	-0.25579	0.00245	Uiso	1.00
C72	C	0.39712	1.40856	-0.10326	0.00245	Uiso	1.00
H73	H	0.40995	1.39710	-0.03538	0.02140	Uiso	1.00
H74	H	0.27450	1.38197	-0.16139	0.02140	Uiso	1.00
H75	H	0.34566	1.44828	-0.08351	0.02140	Uiso	1.00
C76	C	0.67983	1.35042	-0.15076	0.00245	Uiso	1.00
H77	H	0.83057	1.34640	-0.17649	0.02140	Uiso	1.00
H78	H	0.56756	1.31912	-0.20549	0.02140	Uiso	1.00
H79	H	0.69709	1.34391	-0.07779	0.02140	Uiso	1.00
C80	C	0.35399	1.48100	-0.24812	0.00245	Uiso	1.00

H81	H	0.33685	1.51396	-0.27690	0.02140	Uiso	1.00
H82	H	0.32418	1.49495	-0.17041	0.02140	Uiso	1.00
H83	H	0.23247	1.44936	-0.29319	0.02140	Uiso	1.00
C84	C	0.61558	1.45491	-0.36195	0.00245	Uiso	1.00
H85	H	0.77146	1.44181	-0.36984	0.02140	Uiso	1.00
H86	H	0.60819	1.49230	-0.37689	0.02140	Uiso	1.00
H87	H	0.50158	1.42547	-0.41994	0.02140	Uiso	1.00
N88	N	0.14464	0.68632	-0.29577	0.00040	Uiso	1.00
H89	H	0.00129	0.69574	-0.32374	0.00500	Uiso	1.00
C90	C	0.13876	0.62773	-0.32765	0.00245	Uiso	1.00
C91	C	0.02885	0.60601	-0.25852	0.00245	Uiso	1.00
H92	H	0.04467	0.56353	-0.27786	0.01690	Uiso	1.00
H93	H	-0.13960	0.61010	-0.27517	0.01690	Uiso	1.00
C94	C	0.10859	0.63501	-0.14619	0.00245	Uiso	1.00
H95	H	0.01970	0.61943	-0.10058	0.01690	Uiso	1.00
H96	H	0.27197	0.62765	-0.12521	0.01690	Uiso	1.00
C97	C	0.08857	0.69403	-0.12096	0.00245	Uiso	1.00
H98	H	-0.07845	0.70031	-0.13365	0.01690	Uiso	1.00
H99	H	0.14918	0.71551	-0.04050	0.01690	Uiso	1.00
C100	C	0.19945	0.71840	-0.18630	0.00245	Uiso	1.00
C101	C	0.01170	0.60671	-0.43501	0.00245	Uiso	1.00
H102	H	0.00416	0.56353	-0.46483	0.02140	Uiso	1.00
H103	H	0.08161	0.62216	-0.48633	0.02140	Uiso	1.00
H104	H	-0.14818	0.61822	-0.43591	0.02140	Uiso	1.00
C105	C	0.36027	0.60968	-0.33100	0.00245	Uiso	1.00
H106	H	0.45024	0.61533	-0.25586	0.02140	Uiso	1.00
H107	H	0.44868	0.63131	-0.36721	0.02140	Uiso	1.00
H108	H	0.35107	0.56754	-0.37501	0.02140	Uiso	1.00
C109	C	0.12332	0.77281	-0.17605	0.00245	Uiso	1.00
H110	H	0.19597	0.79128	-0.22254	0.02140	Uiso	1.00
H111	H	0.16147	0.79906	-0.09706	0.02140	Uiso	1.00
H112	H	-0.04645	0.76974	-0.20049	0.02140	Uiso	1.00
C113	C	0.43768	0.72524	-0.15039	0.00245	Uiso	1.00
H114	H	0.50018	0.68941	-0.14196	0.02140	Uiso	1.00
H115	H	0.47815	0.75653	-0.07651	0.02140	Uiso	1.00
H116	H	0.51826	0.73630	-0.20382	0.02140	Uiso	1.00
N117	N	0.56307	0.91834	-0.24399	0.00040	Uiso	1.00
H118	H	0.69824	0.90354	-0.26516	0.01800	Uiso	1.00
C119	C	0.57239	0.92138	-0.13675	0.00245	Uiso	1.00
C120	C	0.71968	0.96932	-0.06428	0.00245	Uiso	1.00
H121	H	0.88166	0.96048	-0.07182	0.01690	Uiso	1.00
H122	H	0.70683	0.97412	0.01441	0.01690	Uiso	1.00
C123	C	0.68103	1.02033	-0.08625	0.00245	Uiso	1.00
H124	H	0.52899	1.03337	-0.06863	0.01690	Uiso	1.00
H125	H	0.79782	1.05212	-0.03613	0.01690	Uiso	1.00
C126	C	0.69051	1.01335	-0.19621	0.00245	Uiso	1.00
H127	H	0.85090	1.00615	-0.20950	0.01690	Uiso	1.00
H128	H	0.65580	1.04969	-0.21229	0.01690	Uiso	1.00
C129	C	0.54121	0.96690	-0.27154	0.00245	Uiso	1.00
C130	C	0.35242	0.92265	-0.10973	0.00245	Uiso	1.00
H131	H	0.28593	0.96003	-0.10382	0.02140	Uiso	1.00
H132	H	0.35757	0.91686	-0.03649	0.02140	Uiso	1.00
H133	H	0.24544	0.89110	-0.16708	0.02140	Uiso	1.00
C134	C	0.66069	0.87055	-0.12934	0.00245	Uiso	1.00
H135	H	0.67059	0.86956	-0.05227	0.02140	Uiso	1.00

H136	H	0.81795	0.86762	-0.14853	0.02140	Uiso	1.00
H137	H	0.56105	0.83566	-0.18094	0.02140	Uiso	1.00
C138	C	0.31357	0.98080	-0.27863	0.00245	Uiso	1.00
H139	H	0.20574	0.94528	-0.31761	0.02140	Uiso	1.00
H140	H	0.28893	1.00793	-0.32133	0.02140	Uiso	1.00
H141	H	0.27095	1.00008	-0.20455	0.02140	Uiso	1.00
C142	C	0.60222	0.95392	-0.37617	0.00245	Uiso	1.00
H143	H	0.76224	0.94225	-0.37467	0.02140	Uiso	1.00
H144	H	0.59462	0.98841	-0.40097	0.02140	Uiso	1.00
H145	H	0.49816	0.92148	-0.43294	0.02140	Uiso	1.00
N146	N	-0.08786	0.85838	0.30821	0.00040	Uiso	1.00
H147	H	0.05662	0.84781	0.33157	0.01300	Uiso	1.00
C148	C	-0.08191	0.91697	0.34955	0.00245	Uiso	1.00
C149	C	0.02554	0.94322	0.28594	0.00245	Uiso	1.00
H150	H	0.19432	0.93901	0.30042	0.01690	Uiso	1.00
H151	H	0.00882	0.98579	0.31224	0.01690	Uiso	1.00
C152	C	-0.05613	0.91859	0.17257	0.00245	Uiso	1.00
H153	H	0.03089	0.93716	0.13056	0.01690	Uiso	1.00
H154	H	-0.21984	0.92653	0.15460	0.01690	Uiso	1.00
C155	C	-0.03584	0.85932	0.13775	0.00245	Uiso	1.00
H156	H	0.13123	0.85286	0.14873	0.01690	Uiso	1.00
H157	H	-0.09624	0.84094	0.05634	0.01690	Uiso	1.00
C158	C	-0.14656	0.83083	0.19687	0.00245	Uiso	1.00
C159	C	-0.30304	0.93452	0.35752	0.00245	Uiso	1.00
H160	H	-0.39638	0.93103	0.28420	0.02140	Uiso	1.00
H161	H	-0.29332	0.97600	0.40579	0.02140	Uiso	1.00
H162	H	-0.38809	0.91084	0.39122	0.02140	Uiso	1.00
C163	C	0.04681	0.93351	0.45635	0.00245	Uiso	1.00
H164	H	-0.02253	0.91492	0.50352	0.02140	Uiso	1.00
H165	H	0.05562	0.97641	0.49296	0.02140	Uiso	1.00
H166	H	0.20639	0.92200	0.45424	0.02140	Uiso	1.00
C167	C	-0.07305	0.77553	0.17656	0.00245	Uiso	1.00
H168	H	0.09666	0.77768	0.20007	0.02140	Uiso	1.00
H169	H	-0.11224	0.75242	0.09573	0.02140	Uiso	1.00
H170	H	-0.14670	0.75424	0.21831	0.02140	Uiso	1.00
C171	C	-0.38510	0.82545	0.16264	0.00245	Uiso	1.00
H172	H	-0.42809	0.79603	0.08673	0.02140	Uiso	1.00
H173	H	-0.44582	0.86232	0.15913	0.02140	Uiso	1.00
H174	H	-0.46551	0.81243	0.21392	0.02140	Uiso	1.00
N175	N	0.47913	0.63490	0.27071	0.00040	Uiso	1.00
H176	H	0.35404	0.65222	0.30013	0.00400	Uiso	1.00
C177	C	0.50228	0.58549	0.29557	0.00245	Uiso	1.00
C178	C	0.34215	0.54042	0.22374	0.00245	Uiso	1.00
H179	H	0.18664	0.54890	0.24309	0.01690	Uiso	1.00
H180	H	0.37733	0.50358	0.23775	0.01690	Uiso	1.00
C181	C	0.33522	0.53388	0.11275	0.00245	Uiso	1.00
H182	H	0.48235	0.52004	0.08940	0.01690	Uiso	1.00
H183	H	0.21261	0.50287	0.06533	0.01690	Uiso	1.00
C184	C	0.29465	0.58561	0.09417	0.00245	Uiso	1.00
H185	H	0.13688	0.59554	0.10839	0.01690	Uiso	1.00
H186	H	0.29521	0.58125	0.01454	0.01690	Uiso	1.00
C187	C	0.45200	0.63236	0.16305	0.00245	Uiso	1.00
C188	C	0.45827	0.59784	0.40313	0.00245	Uiso	1.00
H189	H	0.56889	0.62952	0.45768	0.02140	Uiso	1.00
H190	H	0.46968	0.56272	0.42590	0.02140	Uiso	1.00

H191	H	0.30043	0.61024	0.40762	0.02140	Uiso	1.00
C192	C	0.72622	0.56910	0.29392	0.00245	Uiso	1.00
H193	H	0.75159	0.54037	0.33307	0.02140	Uiso	1.00
H194	H	0.84144	0.60314	0.33269	0.02140	Uiso	1.00
H195	H	0.75820	0.55067	0.21732	0.02140	Uiso	1.00
C196	C	0.36510	0.68404	0.16021	0.00245	Uiso	1.00
H197	H	0.21381	0.68786	0.18549	0.02140	Uiso	1.00
H198	H	0.34369	0.68548	0.08312	0.02140	Uiso	1.00
H199	H	0.47137	0.71824	0.20967	0.02140	Uiso	1.00
C200	C	0.66322	0.62939	0.12669	0.00245	Uiso	1.00
H201	H	0.77807	0.66058	0.17975	0.02140	Uiso	1.00
H202	H	0.64496	0.63446	0.05237	0.02140	Uiso	1.00
H203	H	0.72827	0.59170	0.11895	0.02140	Uiso	1.00
N204	N	-0.08766	1.36293	0.32202	0.00040	Uiso	1.00
H205	H	0.05566	1.35062	0.34152	0.00600	Uiso	1.00
C206	C	-0.07256	1.42138	0.37197	0.00245	Uiso	1.00
C207	C	0.04053	1.44996	0.31388	0.00245	Uiso	1.00
H208	H	0.20712	1.44311	0.32529	0.01690	Uiso	1.00
H209	H	0.03397	1.49274	0.34712	0.01690	Uiso	1.00
C210	C	-0.04573	1.43051	0.20075	0.00245	Uiso	1.00
H211	H	-0.20731	1.44072	0.18656	0.01690	Uiso	1.00
H212	H	0.04388	1.45072	0.16242	0.01690	Uiso	1.00
C213	C	-0.03542	1.37110	0.15629	0.00245	Uiso	1.00
H214	H	0.12985	1.36278	0.16379	0.01690	Uiso	1.00
H215	H	-0.09941	1.35647	0.07485	0.01690	Uiso	1.00
C216	C	-0.14967	1.33996	0.20978	0.00245	Uiso	1.00
C217	C	-0.29015	1.44059	0.38337	0.00245	Uiso	1.00
H218	H	-0.38146	1.44090	0.31208	0.02140	Uiso	1.00
H219	H	-0.27470	1.48100	0.43703	0.02140	Uiso	1.00
H220	H	-0.38051	1.41548	0.41219	0.02140	Uiso	1.00
C221	C	0.05756	1.43309	0.47742	0.00245	Uiso	1.00
H222	H	-0.01317	1.41223	0.52063	0.02140	Uiso	1.00
H223	H	0.07182	1.47546	0.52018	0.02140	Uiso	1.00
H224	H	0.21512	1.42069	0.47201	0.02140	Uiso	1.00
C225	C	-0.08374	1.28373	0.18050	0.00245	Uiso	1.00
H226	H	0.08523	1.28417	0.20382	0.02140	Uiso	1.00
H227	H	-0.12267	1.26382	0.09837	0.02140	Uiso	1.00
H228	H	-0.16221	1.26026	0.21690	0.02140	Uiso	1.00
C229	C	-0.38789	1.33729	0.17724	0.00245	Uiso	1.00
H230	H	-0.47027	1.32145	0.22436	0.02140	Uiso	1.00
H231	H	-0.43403	1.31138	0.09831	0.02140	Uiso	1.00
H232	H	-0.44311	1.37581	0.18200	0.02140	Uiso	1.00
O233	O	-0.29909	1.31463	0.42656	0.00500	Uiso	1.00
O234	O	0.77193	1.21303	0.40821	0.00500	Uiso	1.00
O235	O	0.18046	1.21134	0.40180	0.00500	Uiso	1.00
O236	O	0.28368	1.31396	0.41764	0.00500	Uiso	1.00
O237	O	0.86756	1.33899	-0.37027	0.00500	Uiso	1.00
O238	O	-0.23713	1.23535	-0.39107	0.00500	Uiso	1.00
O239	O	0.34669	1.23554	-0.39951	0.00500	Uiso	1.00
O240	O	0.27636	1.33760	-0.37814	0.00500	Uiso	1.00
H241	H	-0.22182	1.33097	0.38544	0.03100	Uiso	1.00
H242	H	-0.26839	1.27712	0.41063	0.04200	Uiso	1.00
H243	H	0.65848	1.18525	0.36083	0.00400	Uiso	1.00
H244	H	0.76105	1.21670	0.47954	0.03100	Uiso	1.00
H245	H	0.02597	1.20920	0.39886	0.05000	Uiso	1.00

H246	H	0.21657	1.24730	0.40005	0.03300	Uiso	1.00
H247	H	0.27825	1.32686	0.49105	0.01100	Uiso	1.00
H248	H	1.02177	1.34118	-0.36843	0.00400	Uiso	1.00
H249	H	0.80681	1.33445	-0.44140	0.01000	Uiso	1.00
H250	H	-0.38996	1.23408	-0.38968	0.00300	Uiso	1.00
H251	H	-0.19422	1.27346	-0.37541	0.01100	Uiso	1.00
H252	H	0.26763	1.21679	-0.36277	0.00400	Uiso	1.00
H253	H	0.28068	1.22232	-0.47270	0.04100	Uiso	1.00
H254	H	0.38945	1.36421	-0.32702	0.02100	Uiso	1.00
H255	H	0.30951	1.30196	-0.37722	0.02200	Uiso	1.00
O256	O	-0.30402	0.80971	0.40952	0.00500	Uiso	1.00
O257	O	0.78182	0.70930	0.39323	0.00500	Uiso	1.00
O258	O	0.19081	0.70832	0.38897	0.00500	Uiso	1.00
O259	O	0.27865	0.81318	0.41092	0.00500	Uiso	1.00
O260	O	0.78407	0.83083	-0.38662	0.00500	Uiso	1.00
O261	O	-0.21568	0.72440	-0.40566	0.00500	Uiso	1.00
O262	O	0.36409	0.72480	-0.41068	0.00500	Uiso	1.00
O263	O	0.34699	0.82818	-0.38463	0.00500	Uiso	1.00
H264	H	-0.22086	0.82899	0.37496	0.02200	Uiso	1.00
H265	H	0.74322	0.74445	0.39156	0.01400	Uiso	1.00
H266	H	0.66736	0.68170	0.34500	0.02000	Uiso	1.00
H267	H	0.03633	0.70578	0.38605	0.00000	Uiso	1.00
H268	H	0.25005	0.71068	0.45913	0.00800	Uiso	1.00
H269	H	0.42878	0.81385	0.40466	0.01800	Uiso	1.00
H270	H	0.23826	0.77502	0.39644	0.01300	Uiso	1.00
H271	H	0.82208	0.79592	-0.38408	0.06700	Uiso	1.00
H272	H	-0.21178	0.71527	-0.47900	0.01400	Uiso	1.00
H273	H	-0.36770	0.72212	-0.40328	0.06400	Uiso	1.00
H274	H	0.28368	0.71056	-0.36753	0.01300	Uiso	1.00
H275	H	0.34807	0.76311	-0.39097	0.03200	Uiso	1.00
H276	H	0.39580	0.86415	-0.33402	0.00400	Uiso	1.00
H277	H	0.75262	0.82252	0.48341	0.00000	Uiso	1.00
H278	H	0.30544	0.83018	0.54654	0.00000	Uiso	1.00
H279	H	0.63201	0.82779	0.61307	0.00000	Uiso	1.00
H280	H	0.43642	0.31548	0.41604	0.00000	Uiso	1.00

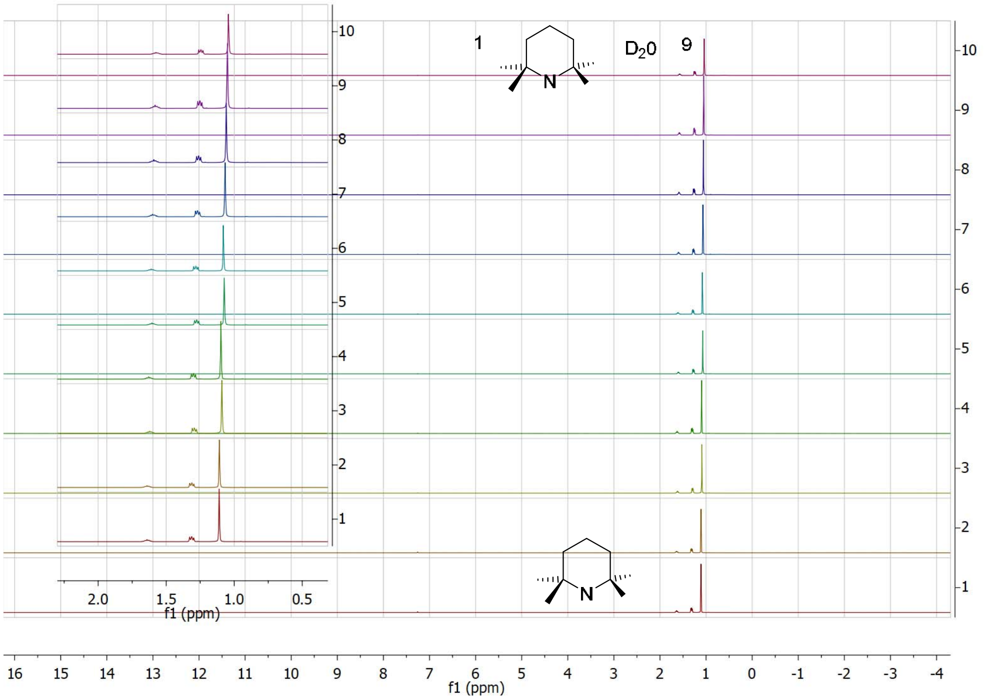

Figure S1 Stacked ^{1}H NMR spectra of TMP:D_2O ratios of 100:0 (spectrum1) to 10:90 (spectrum 10).

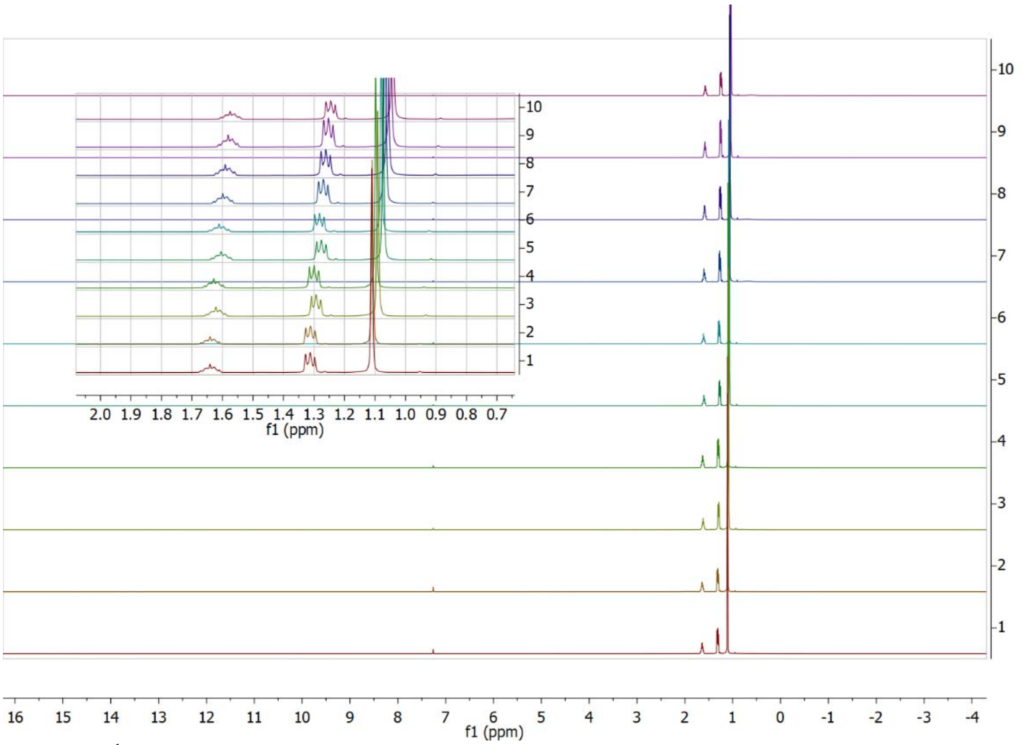

Figure S2 Stacked ^{1}H NMR spectra of TMP:D_2O ratios of 100:0 (spectrum1) to 10:90 (spectrum 10), with expansion to highlight resonance shifts.

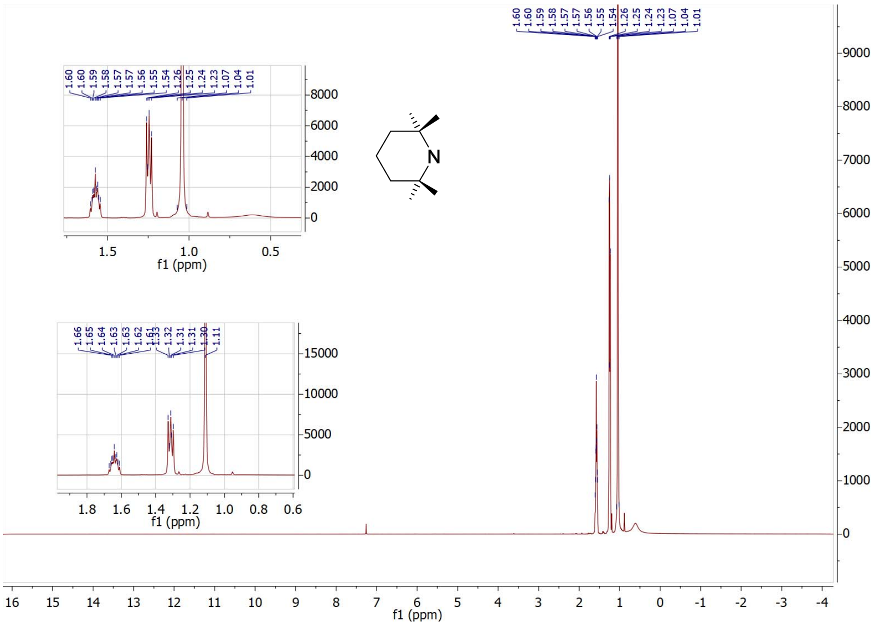

Figure S3 ^{1}H NMR spectrum of 2,2′,6,6′-tetramethylpiperidine for reference.

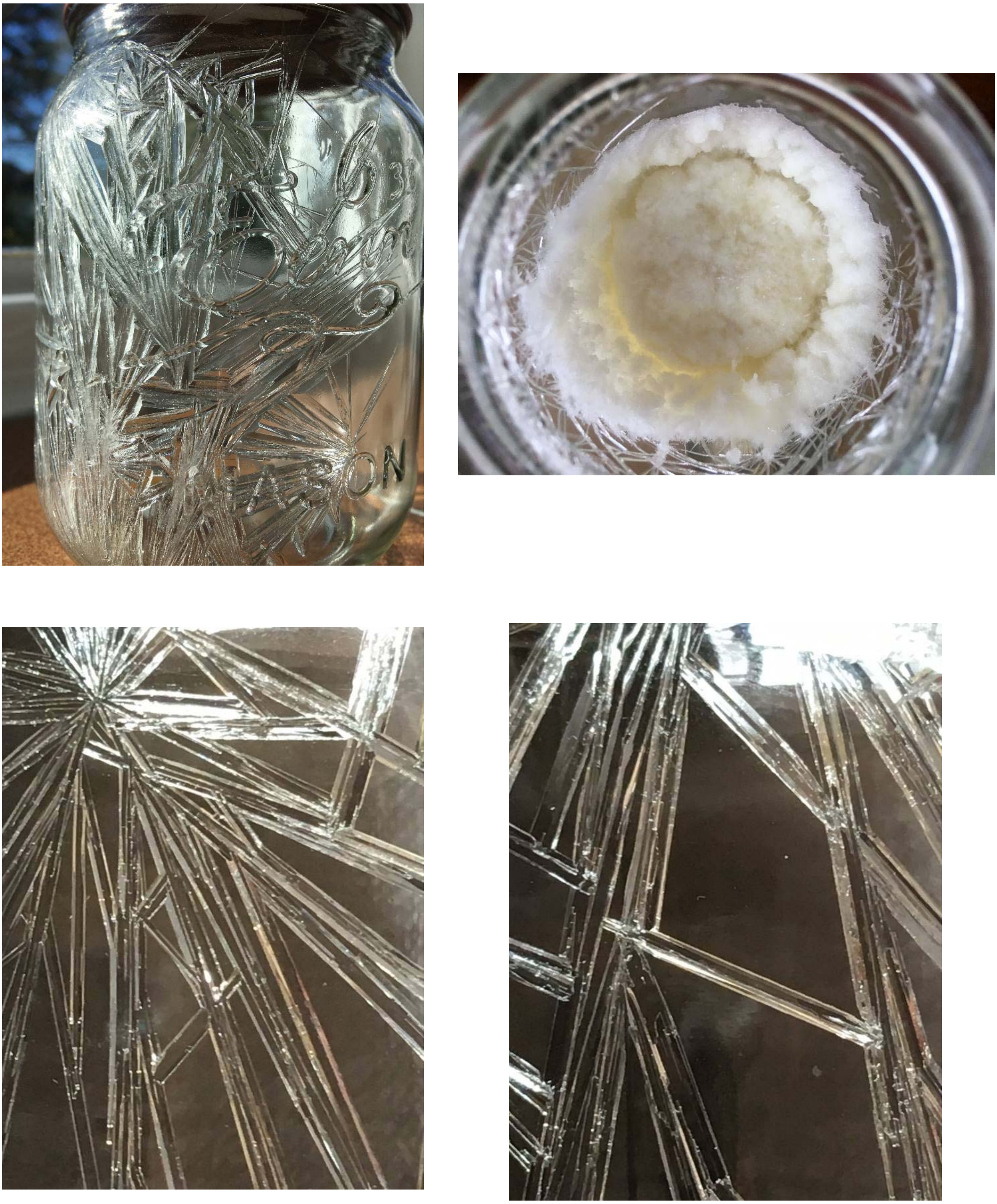

Figure S4 Photographs of the TMP:H_2O complex showing the rod-like morphology.

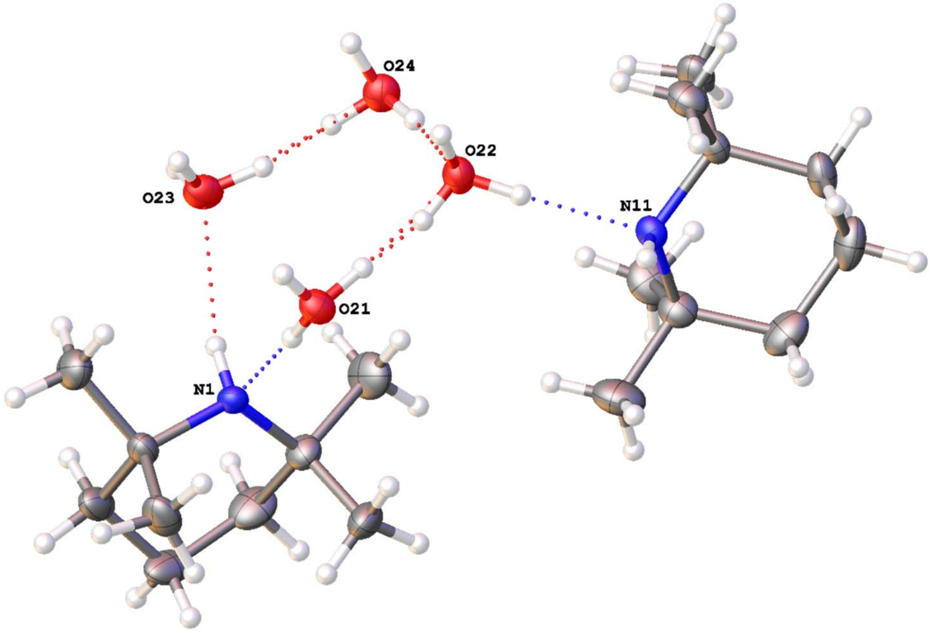

Figure S5 Structure of the water-TMP crystal at 100 K as determined by single crystal X-ray diffraction. Hydrogen atoms are shown at calculated positions and the ellipsoids are drawn at the 90% probability level.

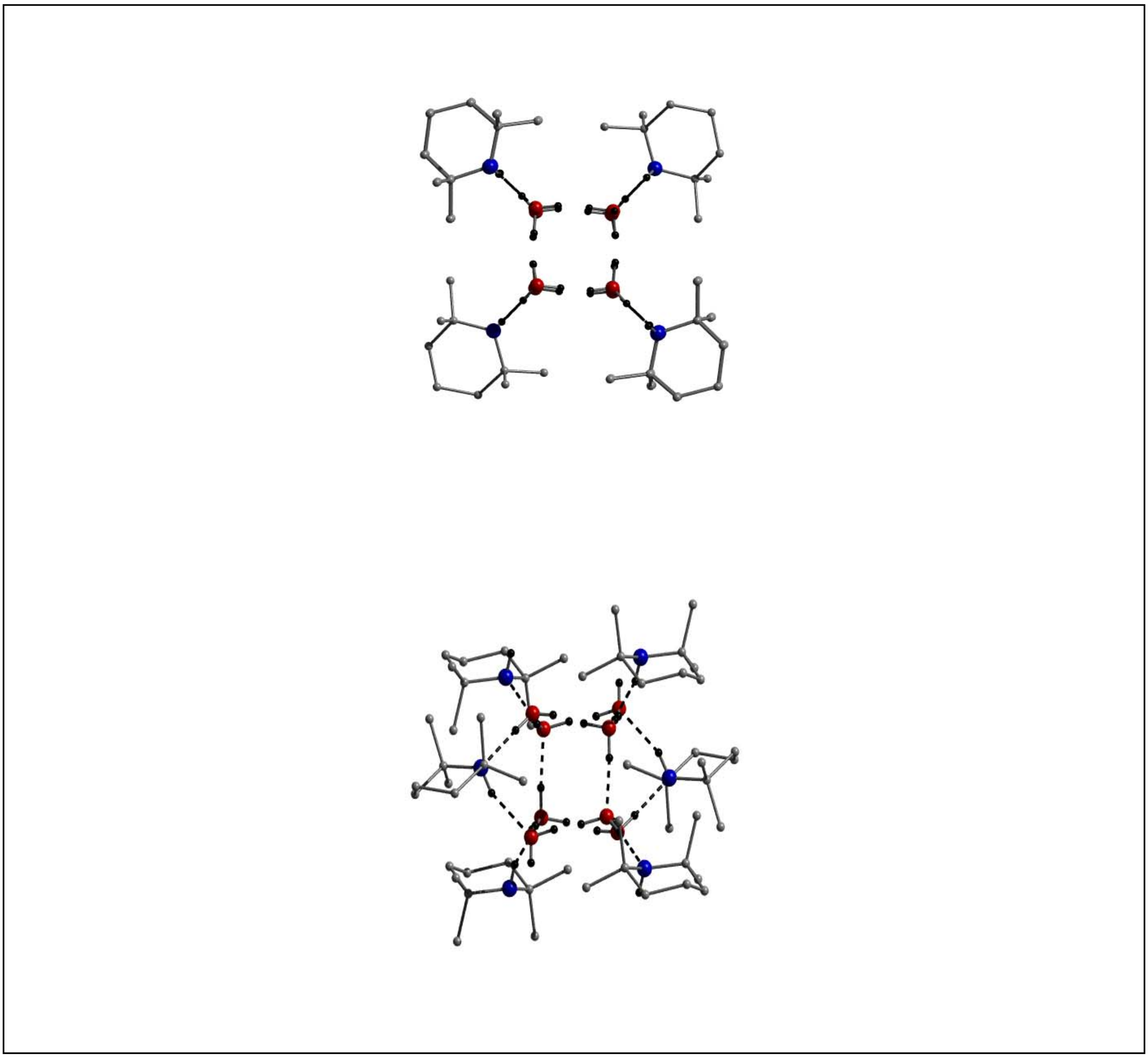

Figure S6 Molecular views of the water channel showing the interaction between the water molecules and external TMP molecules (X-ray data, 100 K). Top: view down the water channel. Bottom: side view.

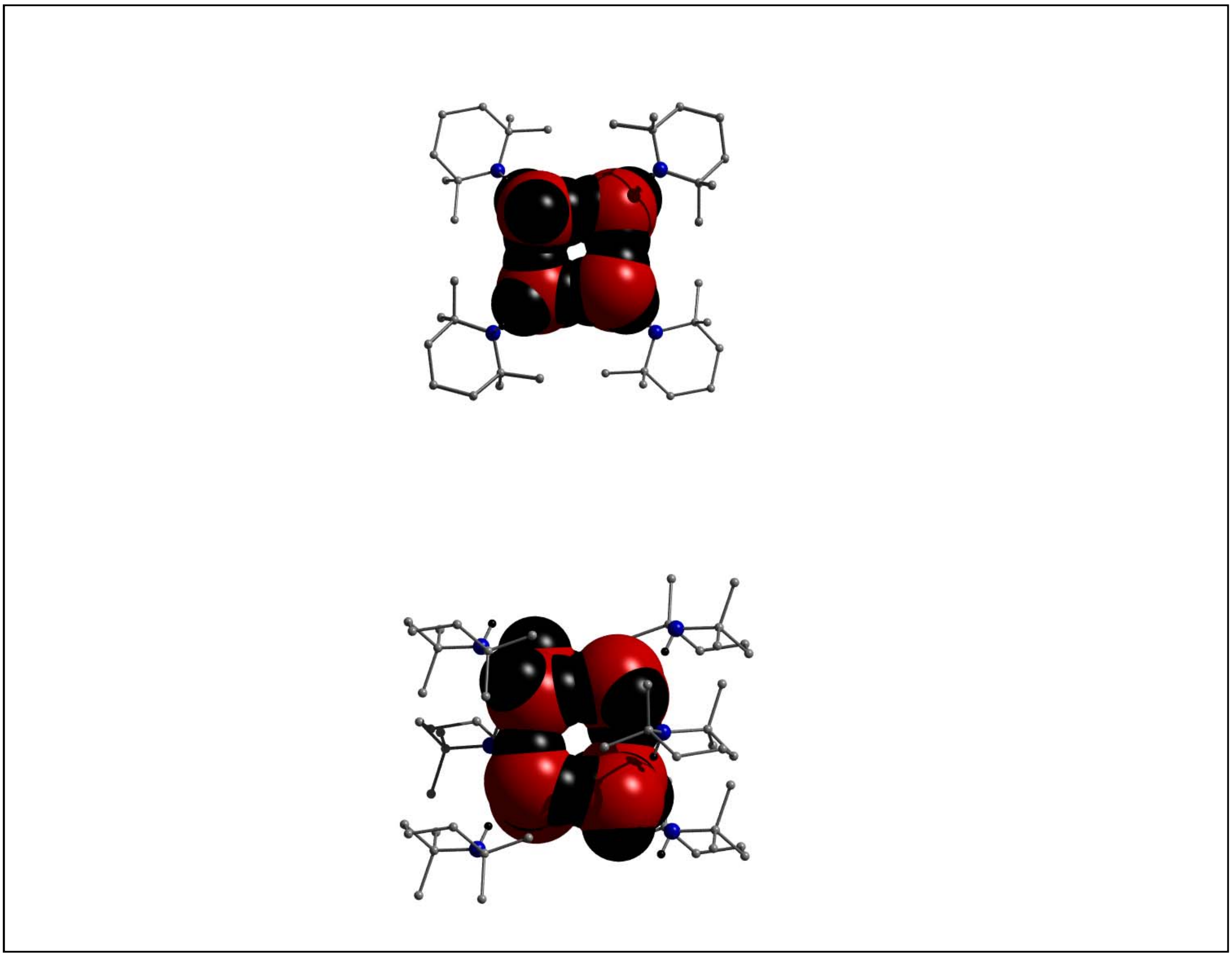

Figure S7 Molecular views of the water channel (as in Figure S7) using space filling models for the water molecules (X-ray data, 100 K). Top: view down the water channel. Bottom: side view.

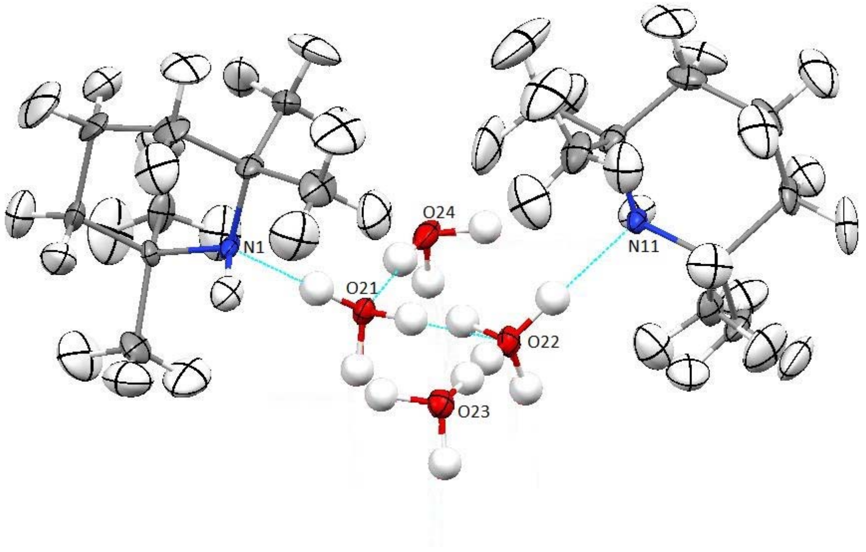

Figure S8 Hydrogen bonds between water and TMP in the crystal at 100 K (neutron data).

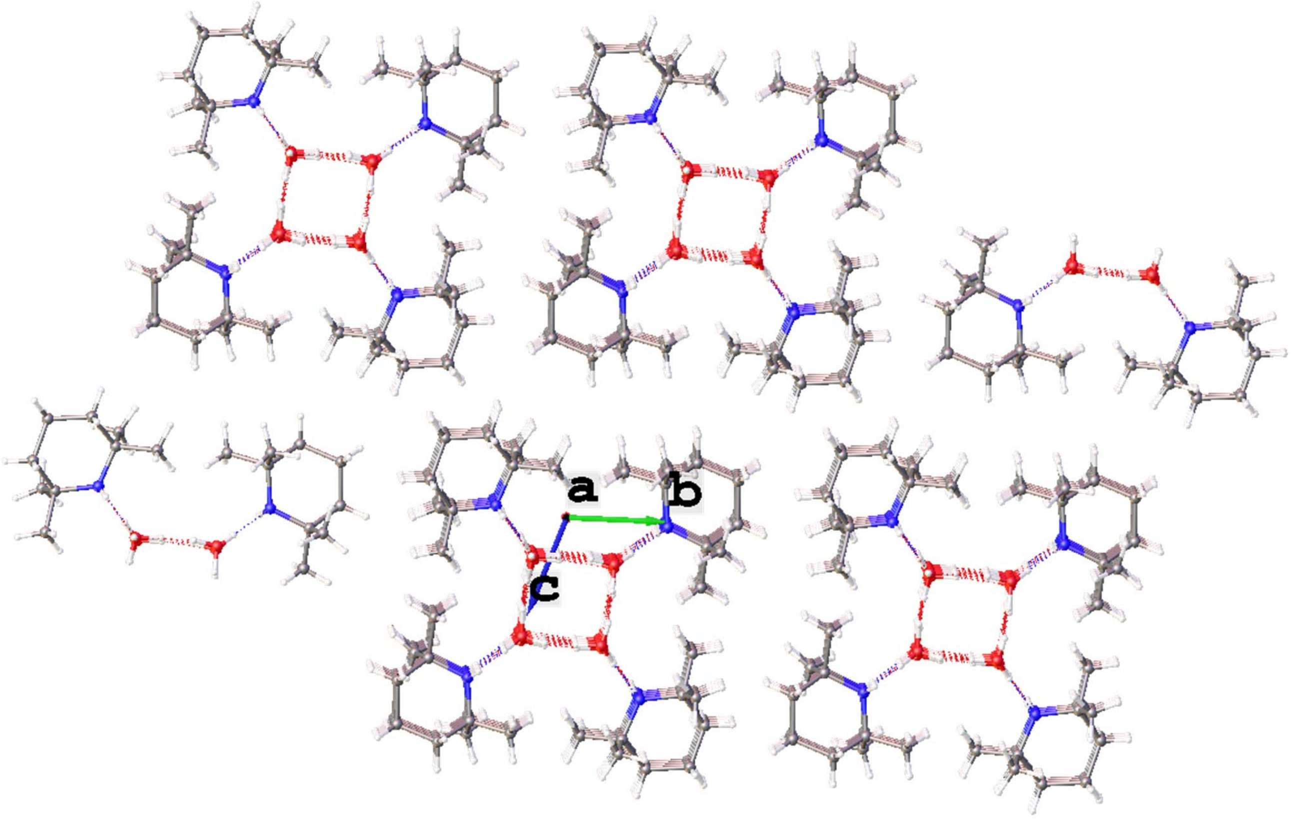

Figure S9 Extended structure showing a view down the molecular water pipe "tubes" (neutron data, 100 K).

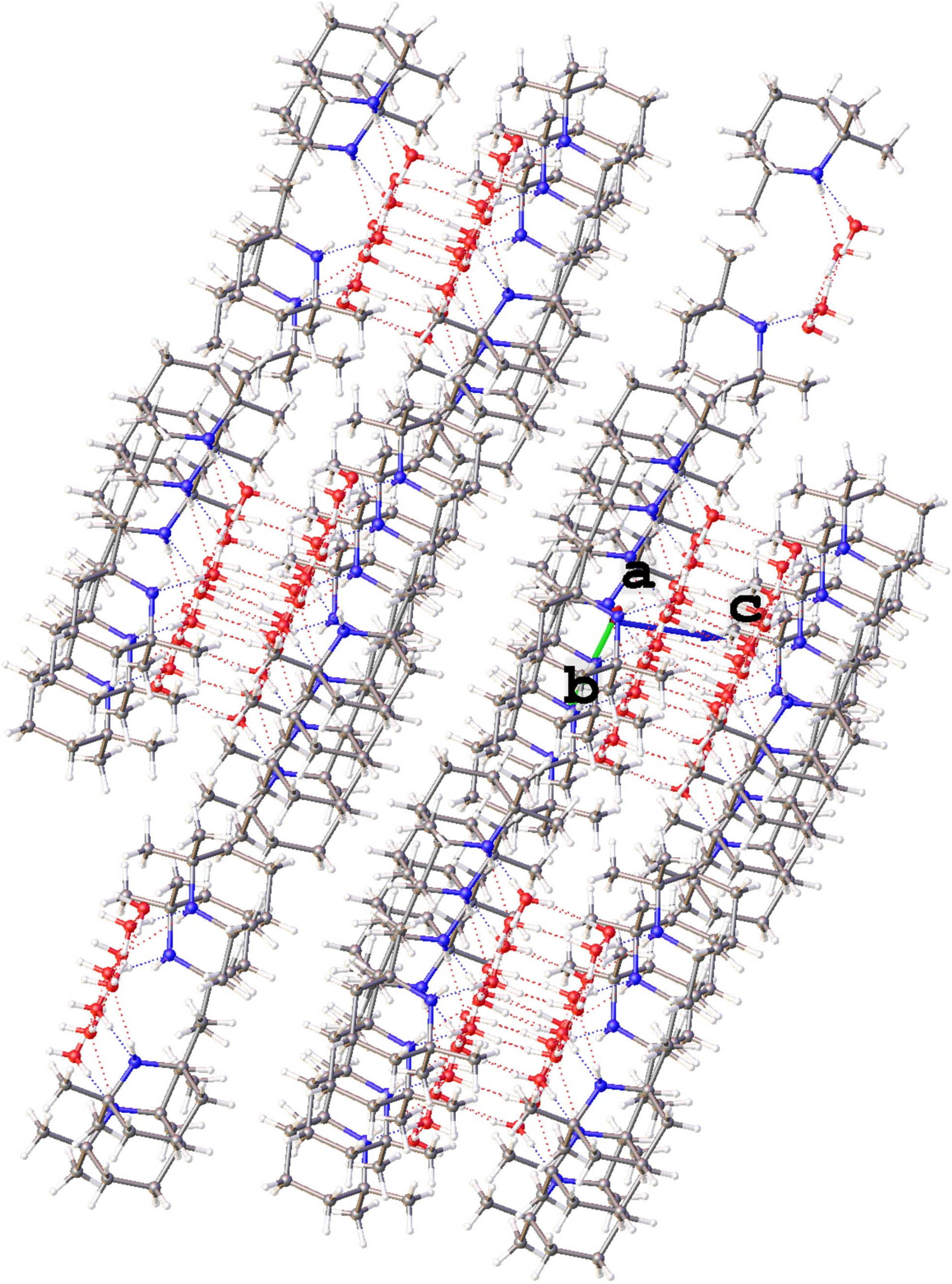

Figure S10 Side view of the crystalline packing showing individual molecular “water pipes” (neutron data, 100 K).

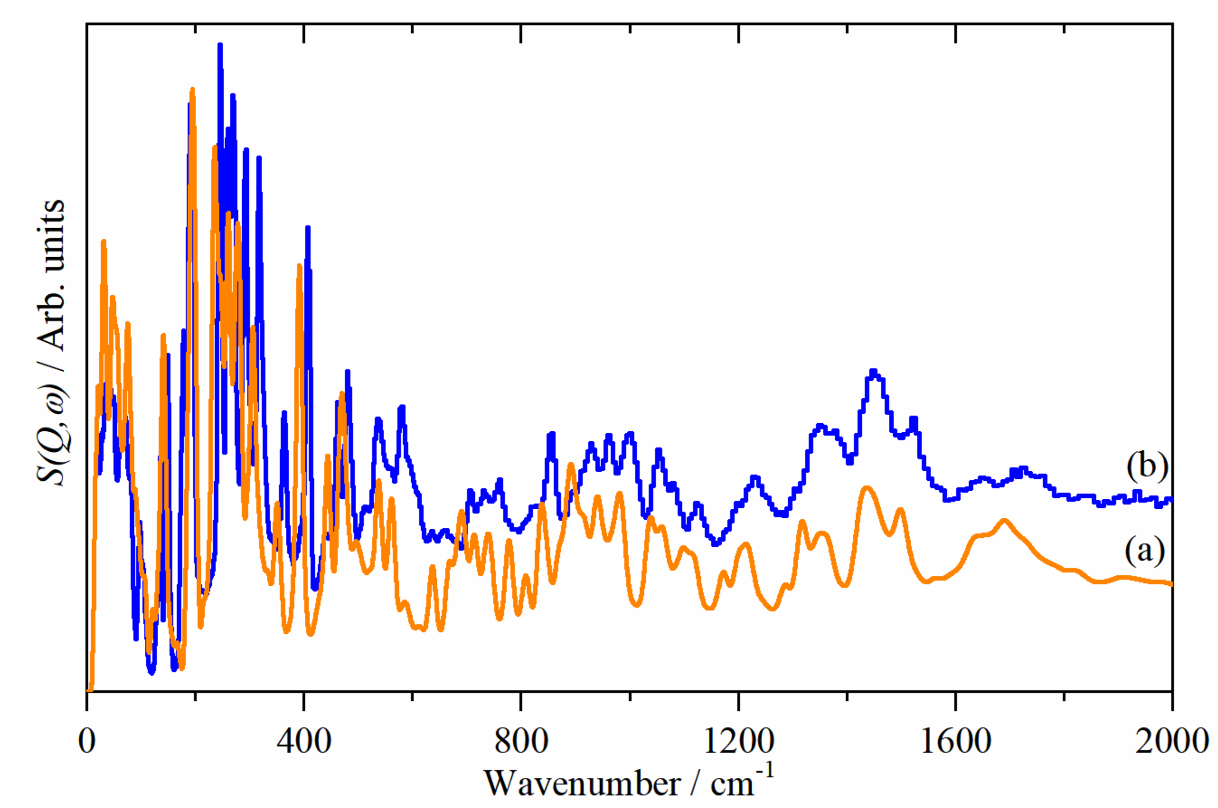

Figure S11 Comparison of: (a) the calculated INS spectrum of TMP:H_2O with (b) the experimental spectrum at 10 K.

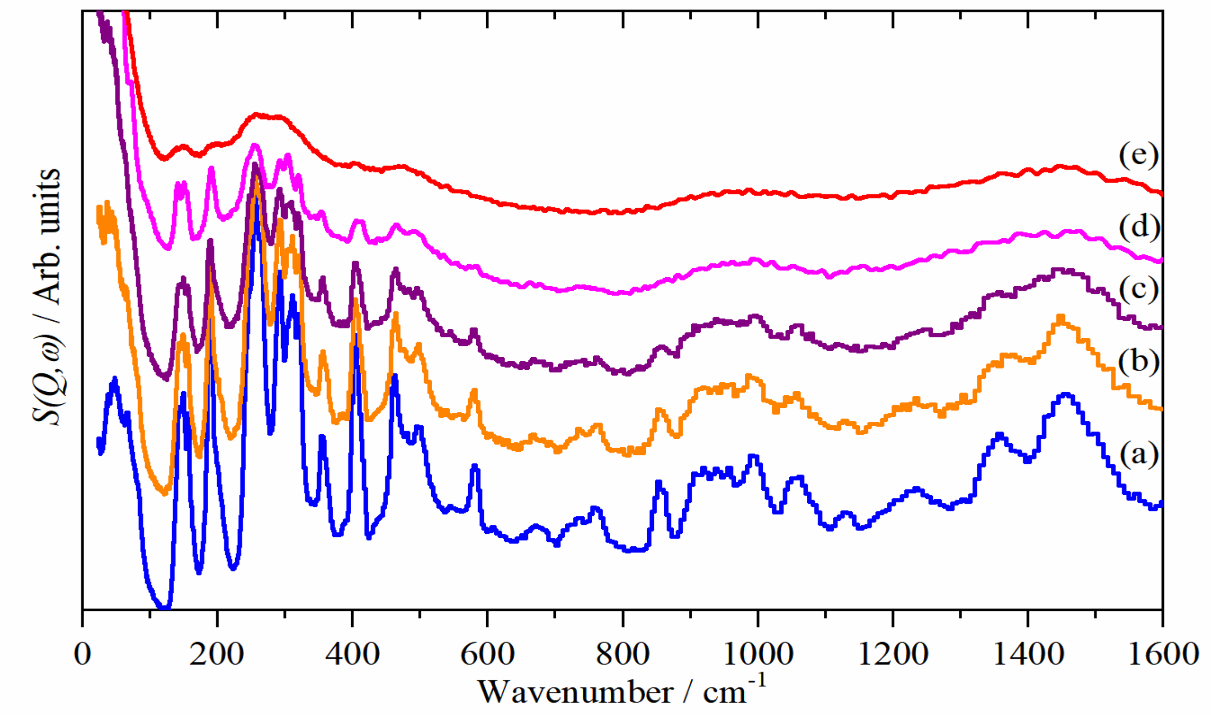

Figure S12 Evolution of the INS spectrum of TMP with temperature. (a) 10 K, (b) 50 K, (c) 100 K, (d) 200 K and (e) 220 K.

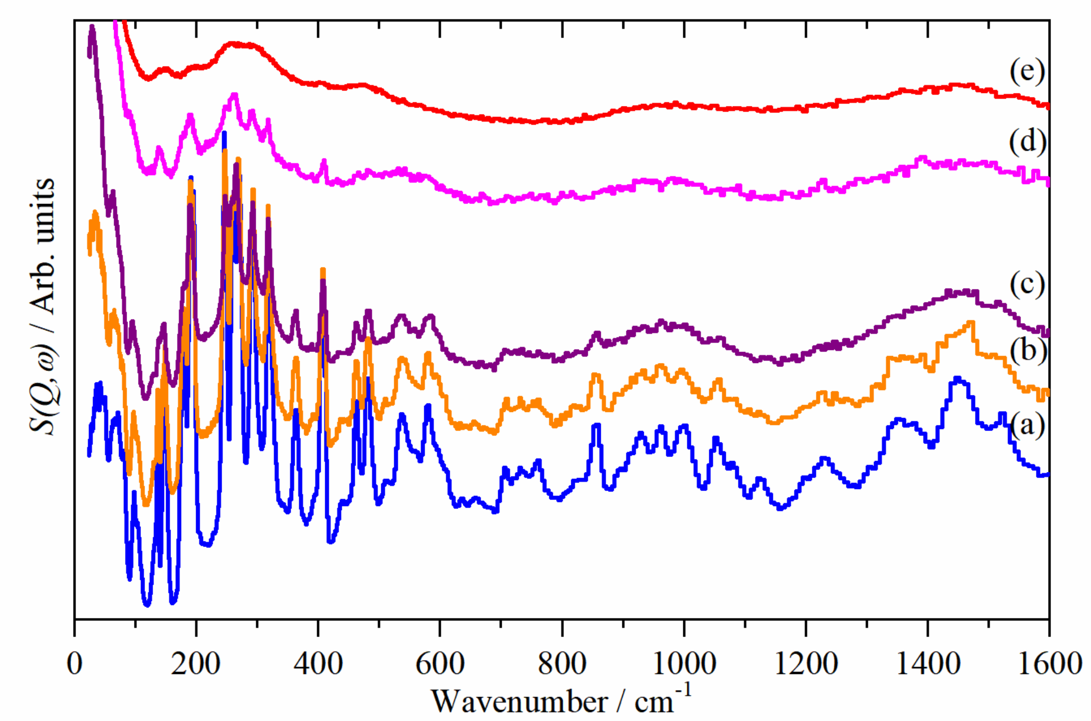

Figure S13 Evolution of the INS spectrum of TMP-H_2O with temperature. (a) 10 K, (b) 50 K, (c) 100 K, (d) 200 K and (e) 220 K.

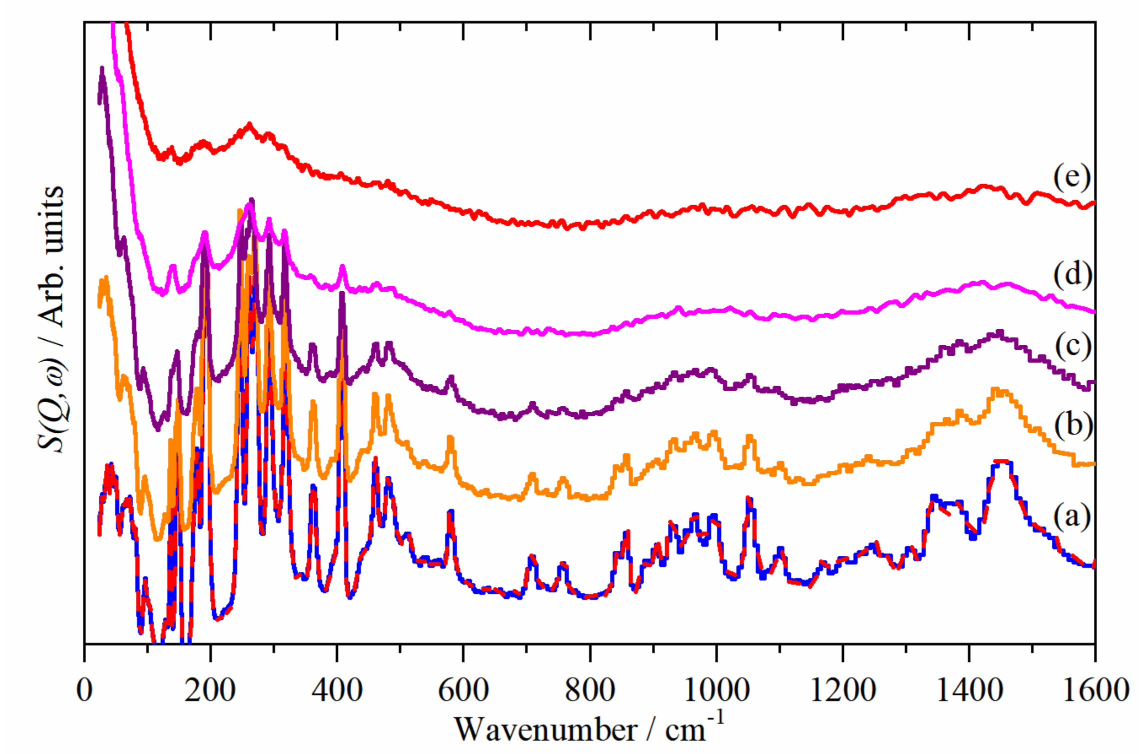

Figure S14 Evolution of the INS spectrum of TMP-D_2O with temperature. (a) 11 K, (b) 50 K, (c) 100 K, (d) 200 K and (e) 220 K. In (a) the solid blue line is the initial spectrum, the dashed red line is the sample after heating to 220 K and cooling back
